# A History of Drug Discovery for Treatment of Nausea and Vomiting and the Implications for Future Research

**DOI:** 10.3389/fphar.2018.00913

**Published:** 2018-09-04

**Authors:** Gareth J. Sanger, Paul L. R. Andrews

**Affiliations:** ^1^Blizard Institute and the National Centre for Bowel Research, Barts and The London School of Medicine and Dentistry, Queen Mary University of London, London, United Kingdom; ^2^Division of Biomedical Sciences, St George's University of London, London, United Kingdom

**Keywords:** nausea and vomiting, drug discovery, metoclopramide, histamine H_1_ receptor antagonists, muscarinic receptor antagonists, 5-hydroxytryptamine_3_ receptor antagonists, neurokinin_1_ receptor antagonists, olanzapine

## Abstract

The origins of the major classes of current anti-emetics are examined. Serendipity is a recurrent theme in discovery of their anti-emetic properties and repurposing from one indication to another is a continuing trend. Notably, the discoveries have occurred against a background of company mergers and changing anti-emetic requirements. Major drug classes include: (i) *Muscarinic receptor antagonists*–originated from historical accounts of plant extracts containing atropine and hyoscine with development stimulated by the need to prevent sea-sickness among soldiers during beach landings; (ii) *Histamine receptor antagonists*–searching for replacements for the anti-malaria drug quinine, in short supply because of wartime shipping blockade, facilitated the discovery of histamine (H_1_) antagonists (e.g., dimenhydrinate), followed by serendipitous discovery of anti-emetic activity against motion sickness in a patient undergoing treatment for urticaria; (iii) *Phenothiazines and dopamine receptor antagonists*–investigations of their pharmacology as “sedatives” (e.g., chlorpromazine) implicated dopamine receptors in emesis, leading to development of selective dopamine (D_2_) receptor antagonists (e.g., domperidone with poor ability to penetrate the blood-brain barrier) as anti-emetics in chemotherapy and surgery; (iv) *Metoclopramide and selective 5-hydroxytryptamine*_3_ *(5-HT*_3_*) receptor antagonists–*metoclopramide was initially assumed to act only via D_2_ receptor antagonism but subsequently its gastric motility stimulant effect (proposed to contribute to the anti-emetic action) was shown to be due to 5-hydroxytryptamine_4_ receptor agonism. Pre-clinical studies showed that anti-emetic efficacy against the newly-introduced, highly emetic, chemotherapeutic agent cisplatin was due to antagonism at 5-HT_3_ receptors. The latter led to identification of selective 5-HT_3_ receptor antagonists (e.g., granisetron), a major breakthrough in treatment of chemotherapy-induced emesis; (v) *Neurokinin*_1_ *receptor antagonists*–antagonists of the actions of substance P were developed as analgesics but pre-clinical studies identified broad-spectrum anti-emetic effects; clinical studies showed particular efficacy in the delayed phase of chemotherapy-induced emesis. Finally, the repurposing of different drugs for treatment of nausea and vomiting is examined, particularly during palliative care, and also the challenges in identifying novel anti-emetic drugs, particularly for treatment of nausea as compared to vomiting. We consider the lessons from the past for the future and ask why there has not been a major breakthrough in the last 20 years.

## Introduction

**Figure 1:**
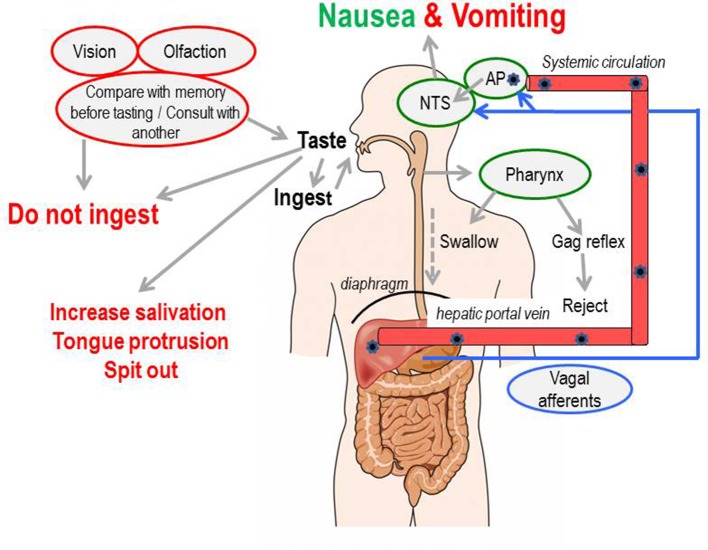
A summary of the levels of defense employed to initially avoid and, if required, to detect and respond to toxins ingested with the food. AP, area postrema (also known as the “chemoreceptor trigger zone” for emesis, but see text for discussion); NTS, nucleus tractus solitarius; the site in the dorsal brainstem where inputs from the vagal afferents and the area postrema are integrated and from which outputs pass to other areas of the brainstem to coordinate the motor outputs for vomiting and from which information is relayed to “higher” brain regions to evoke the sensation of nausea. Figure adapted and modified from Andrews (1993).

The sensation of nausea and the ability to vomit are key components of human defenses against unintentional ingestion of noxious material and are part of a hierarchically organized defensive system (Figure 1; Davis et al., 1986; Stern et al., 2011). Ingested toxins must be detected rapidly and reliably, nausea induced quickly to limit further ingestion, and vomiting initiated promptly to void contaminated ingested material whilst still in the lumen of the upper digestive tract.

Nausea is considered a “warning.” It can be considered to represent “low intensity” stimulation of afferent pathways, which if activated more intensely, trigger vomiting, yet paradoxically, it is considered easier to prevent vomiting rather than nausea by anti-emetic drugs (Andrews and Sanger, 2014). Likewise, risk factors for induction of nausea as opposed to vomiting may also differ, as exemplified by post-operative nausea and vomiting (Stadler et al., 2003). An accepted function of nausea is that it causes a learned aversion to the food associated with the nausea, leading to avoidance when subsequently encountered, sometimes lifelong (Stern et al., 2011).

**Figure 2:**
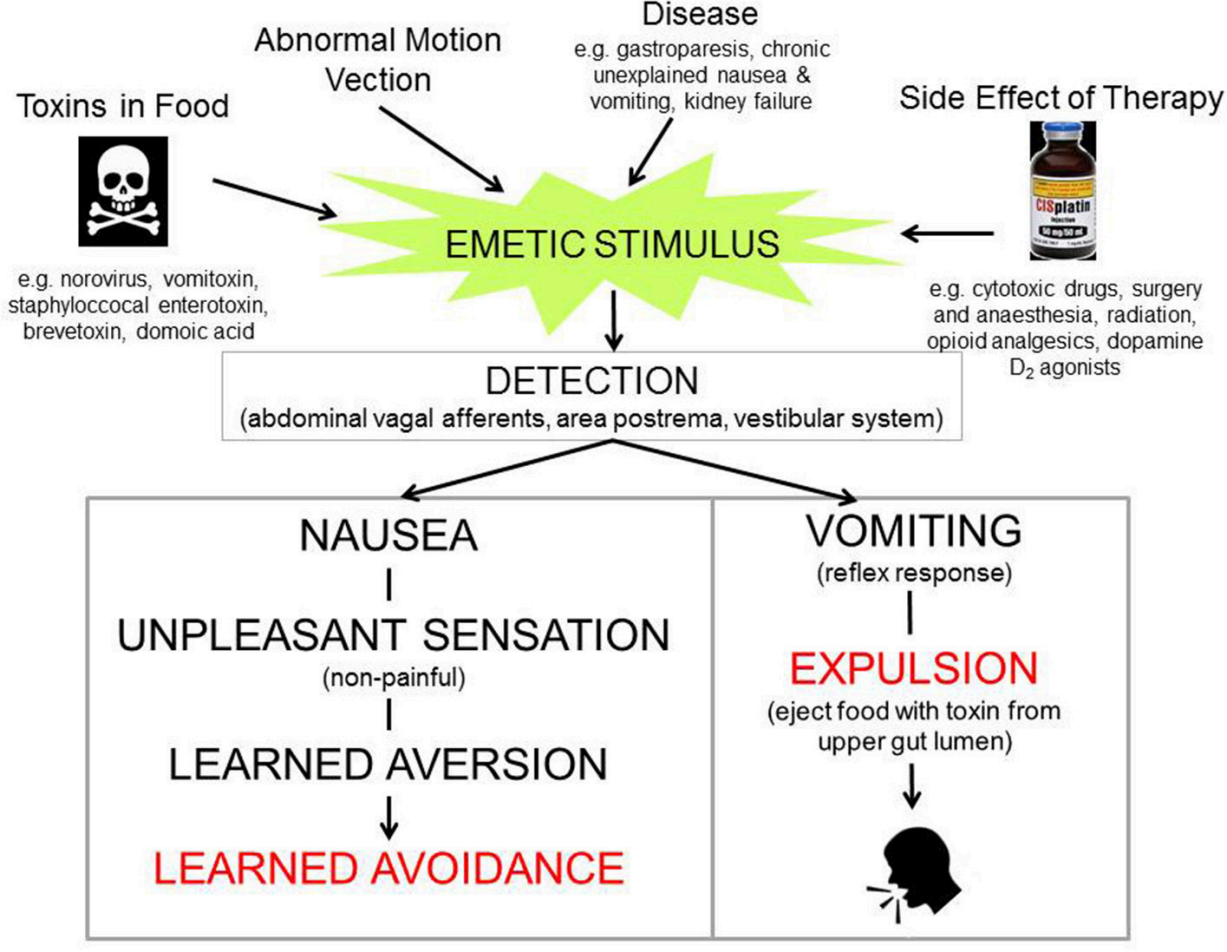
Diagram illustrating that nausea and vomiting can be evoked by stimuli ranging from toxins in the food where they may be viewed as an “appropriate” response helping to defend the animal, to diseases and therapeutic interventions where they are viewed as undesirable and are classified as “symptoms” or “side-effects.” Profile of the head from http://getdrawings.com/talking-head-silhouette.

The pathways which evolved to detect ingested toxins and aberrant motion can also be triggered by diverse diseases and pharmacological therapies (Figure 2). Thus, nausea and vomiting rather than being adaptive responses of evolutionary significance (arguably including pregnancy sickness in humans; Profet, 1988, 1992; Flaxman and Sherman, 2000 but for a different view see Brown et al., 1997; Weigel et al., 2006) become “symptoms of disease” or “side-effects of drugs” which often require treatment (Figure 2). Motion sickness, pregnancy sickness and adverse effects of therapy (primarily for cancer) have driven the development of anti-emetic drugs since the early 1940s.

Anti-emetics are sometimes viewed as a niche therapeutic area but this is incorrect as: (a) Nausea and vomiting are amongst the most common reasons for an emergency department visit (Meek et al., 2015), (b) An anti-emetic (ondansetron) was on the list of drugs with sales of one billion $US a year before patent expiry and together with metoclopramide (an anti-emetic and gastric prokinetic drug), ondansetron has been included on the World Health Organization list of essential medicines^1^, (c) Developments in anti-emetics (particularly antagonists at 5-hydroxytryptamine_3_ receptors; 5-HT_3_) were included in the “top five advances” in modern oncology in a 2014 American Society for Clinical Oncology survey^2^, (d) Anti-emetics decrease overall healthcare costs in cancer patients because they enable treatment in day centers and reduce the need for hospitalization following severe vomiting; a similar argument applies to post-operative nausea and vomiting (PONV), reducing the need for longer (particularly overnight) hospitalization, (e) Anti-emetics provide rare examples of clinical agents acting as an antagonist at a ligand-gated ion channel (5-HT_3_) and at a receptor for a peptide (neurokinin_1_; NK_1_), (f) Significant conditions remain in which nausea represents a defining but poorly-treated symptom in large patient populations (e.g., palliative care, gastroparesis, functional dyspepsia).

**Figure 3:**
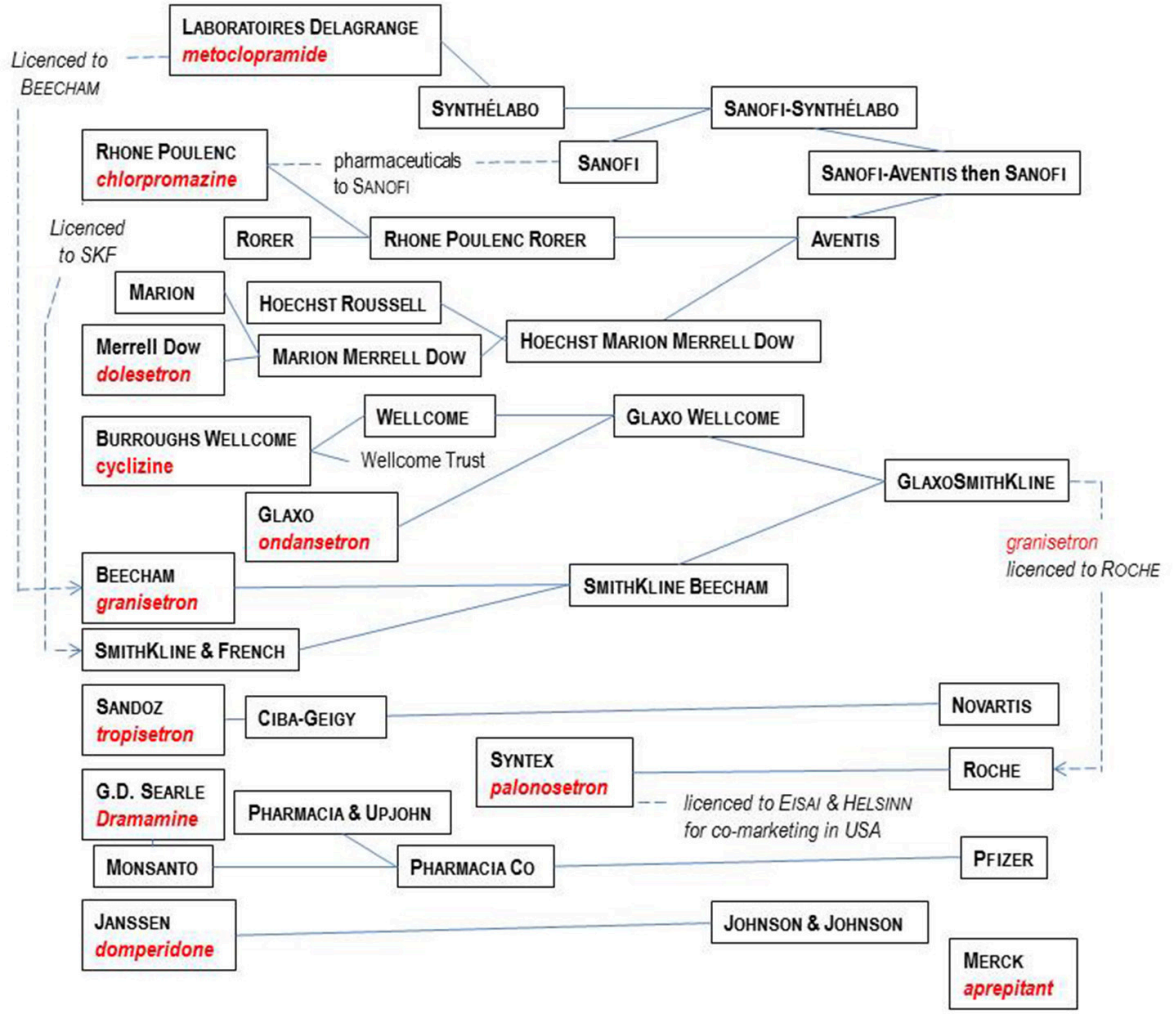
Major players in the pharmaceutical industry responsible for the development of the main anti-emetic drugs over the time course covered by this review. See text for details and references.

In this review, current nomenclature is used^3^ to describe G-Protein Coupled Receptors (GPCRs) and ion channels. Nevertheless, it is important to appreciate that when many anti-emetic drugs were discovered, their target GPCR or ion channel was not fully characterized or even defined. Progress in understanding anti-emetic drug physiology and receptor pharmacology can therefore be viewed as running in parallel with characterization of these targets. Such progress also illustrates the evolution in methods of drug discovery, from early reliance on animals to define therapeutic and adverse effects of drug candidates, through to the use of such models to define novel receptor functions (e.g., 5-HT_3_ receptor) and today's focus on recombinant human receptors to characterize compound activity before translation using animals and humans. The last 30 years in particular, have also seen major re-organizations of the pharmaceutical industry. Figure 3 shows the companies which played significant roles in anti-emetic drug discovery, many of which disappeared during mergers and takeovers, impacting research. Table 1 provides details of key contributions.

**Table 1:** The major pharmaceutical companies involved in the discovery of anti-emetic drugs during the period covered by this review and a summary of their key contribution to the area.

**Rhône-Poulenc Laboratories**
•Rapidly focussed on the therapeutic potential of the newly-discovered “antihistamines,” searching libraries of compounds originally synthesized for another use. The first antihistamine to treat anaphylaxis and allergic reactions was phenbenzamine, introduced into the clinic in 1942. •Re-examination of the antihistamines to optimize the “anti-shock” property led to synthesis in 1946 of chlorpromazine (4560-R.P). This compound had low antihistamine activity but in 1951 the company demonstrated its ability to prevent emesis evoked by apomorphine in dogs.
**G.D. Searle & Co**
•Introduced the “antihistamine” dimenhydrinate (Dramamine), a combination of diphenhydramine and 8-chlorotheophylline (a mild stimulant and derivative of theophylline) as a counter measure against the drowsiness, somnolence, and sedation caused by H_1_ receptor antagonism within the brain.
**Burroughs Wellcome**
•Developed the “antihistamine” cyclizine, in 1947, subsequently taken on the Apollo moon missions as a treatment for space sickness.
**Laboratoires Delagrange**
•Identified metoclopramide in the mid-1950s, during a programme aimed at improving the properties of procainamide, a cardiac anti-arrhythmic and local anesthetic drug derived from procaine. The drug had negligible local anesthetic or cardiac anti-arrhythmic activity but an ability to inhibit emesis in dogs evoked by multiple stimuli. Soon after, metoclopramide was also found to stimulate GI motility and reduce symptoms associated with various upper GI disorders.
**Janssen Pharmaceutica**
•Among the antipsychotic compounds the company had developed in the mid-1950s, some were effective antagonists at the dopamine receptors in the chemoreceptor trigger zone, an area of brain outside the blood-brain barrier, involved in regulation of vomiting. Domperidone was identified in 1974 as an antagonist which did not cross the blood-brain barrier and hence, less likely to evoke the extrapyramidal side-effects.
**Merrell Dow**
•Synthesized MDL72222 from the chemical template of cocaine, the first selective 5-HT_3_ receptor antagonist, originally aimed at the treatment of migraine. A later compound (MDL73147 or dolasetron) was marketed for the control for chemo-radiotherapy-induced emesis.
**Beecham Pharmaceuticals**
•Identified the anti-emetic activity of the 5-HT_3_ receptor antagonists, developing its own molecule (BRL43694 or granisetron, launched by SmithKline Beecham for the control of chemoradiotherapy-induced emesis) and successfully filed a patent to cover the anti-emetic use of Glaxo's compound (GR38032F or ondansetron), originally designed for treatment of “a variety of disorders including migraine” before being specifically patented for treatment of depression, schizophrenia, anxiety, and cognitive disorders.
**Glaxo**
•Identified ondansetron for the treatment of migraine and a variety of CNS disorders. Subsequent marketing as an anti-emetic drug incurred royalty payments to Beecham/SmithKline Beecham who owned the patent covering the anti-emetic use of this drug.
**Sandoz**
•Identified the 5-HT_3_ receptor antagonist ICS 205-930 (tropisetron), originally for treatment of migraine, subsequently sponsoring research to characterize its anti-emetic activity and “re-purpose” for treatment of chemoradiotherapy-induced emesis.
**Merck**
•Aprepitant introduced in 2003, following initial characterization for treatment of depression and emesis and a long history of failure of other NK_1_ receptor antagonists to treat pain.
**Syntex Discovery Research**
•Synthesized and characterized palonosetron (RS 25259-197), licensed to Eisai and Helsinn for co-marketing in the USA in 2003 (the same year as aprepitant).

*See text for further details*.

This review outlines the mechanisms of nausea and vomiting, providing a background to the discovery and pharmacology of licensed anti-emetic drugs and compounds still in clinical development. We examine the shifting strategies adopted by the pharmaceutical industry and academia over the last ~75 years. Lessons learned and challenges to further advances are also highlighted, together with current research trends.

## Common causes of nausea and vomiting

The three main causes of nausea and vomiting which may require therapeutic intervention are diseases (organic and functional), drug or other therapies (e.g., PONV) and motion sickness. Amongst the diseases, digestive tract disorders are currently being investigated most actively, with interest focused on the genesis of nausea in conditions such as gastroparesis (see below). Treatment of the emetic side effects of anti-cancer chemotherapy (Andrews and Rudd, 2016), analgesics in palliative care (Smith and Laufer, 2014) and PONV (Horn et al., 2014) are the commonest examples in the “side-effect of therapy” category (for reviews see Stern et al., 2011; Koch and Hasler, 2017) but it should also be noted that nausea and vomiting are surprisingly common side-effects of drugs in general; the Electronic Medicines Compendium indicates nausea as an adverse event for >50% of a wide range of drugs and both nausea and vomiting for >33% (Lee, 2006). Indeed, as an adverse event, nausea and vomiting is second only to the potential for abuse liability in their impact on development of new chemical entities (NCEs) as therapeutic agents (Holmes et al., 2009), so predicting such liability early in the discovery process is of high importance. Meta-analysis and database mining of “historic” animal and human studies (which may never be repeated) provide a useful approach to identification of chemical templates most likely to induce vomiting (Parkinson et al., 2012; Percie du Sert et al., 2012).

Motion sickness is not a disease but, apart from food poisoning and pregnancy, it is probably the most likely cause of nausea and vomiting experienced by readers of this review. Medications used for travel sickness (e.g., Joy Rides® and Kwells® [formulations of hyoscine hydrobromide]; Stugeron® [cinnarizine]) are rare examples (in the UK) of widely-used anti-emetics available without prescription. Interest in motion sickness continues because of “space motion sickness,” occurring in ~70% of astronauts during the first 3 days in space (Crampton, 1990; Weerts et al., 2015).

## Clinical need for anti-emetic drugs

**Figure 4:**
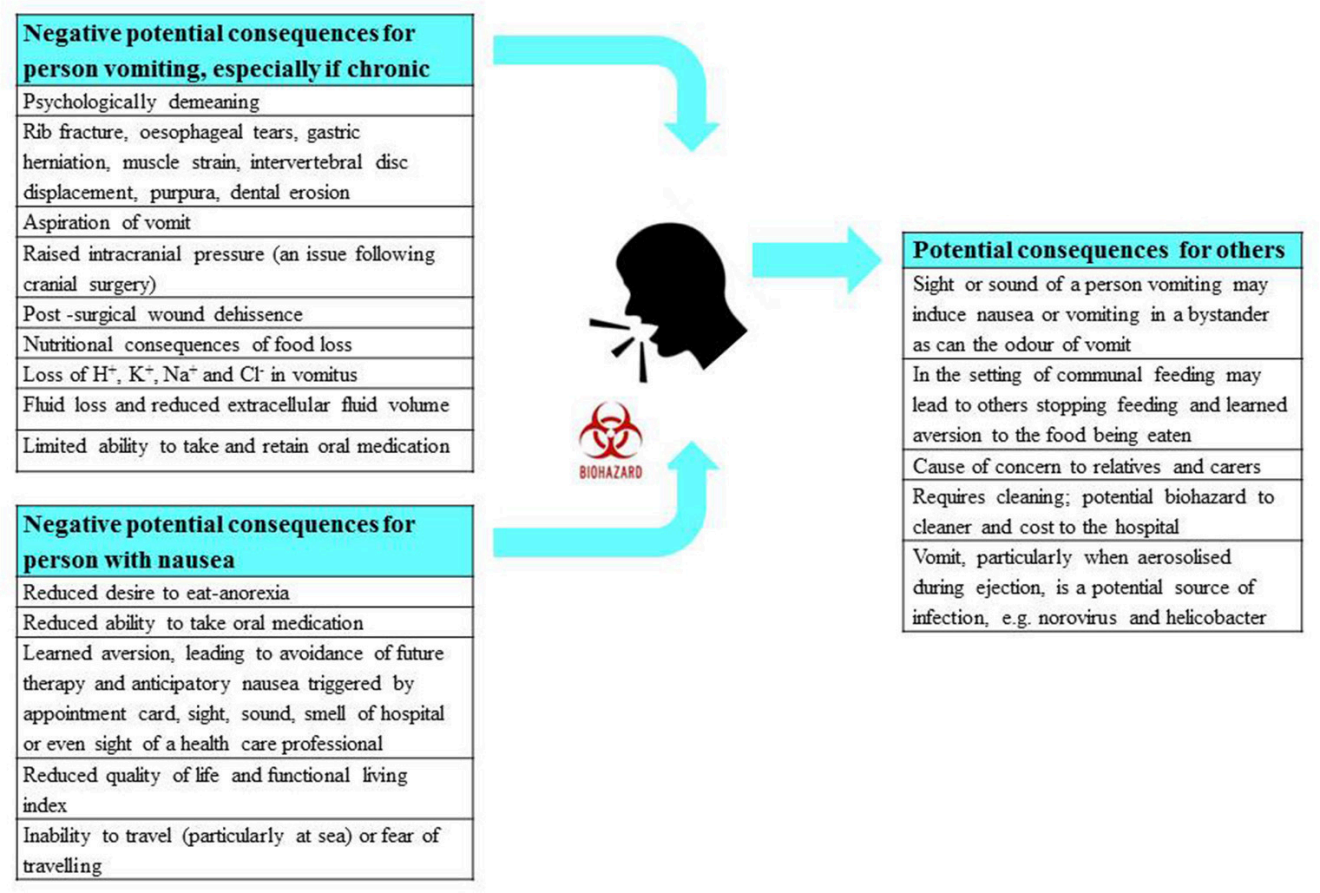
A summary of the physical, physiological, and psychological consequences of nausea and vomiting for the person suffering, as well as for any observers including health care professionals. The potential risk of infection from vomiting is also highlighted. Profile of the head from http://getdrawings.com/talking-head-silhouette.

Vomiting has a diverse range of potential impacts upon the person involved and also potentially on others. The consequences are psychological (e.g., demeaning), physical (e.g., chronic fatigue and fractured ribs), metabolic (e.g., dehydration, anorexia) and when caused by medications, can affect therapeutic outcomes (e.g., if treatments are refused); these are summarized in Figure 4. In circumstances when the vomiting is not induced by food-borne toxins, blockade by an anti-emetic drug is desirable. Notably, although vomiting is unpleasant, patients are frequently more concerned about nausea, because as with chronic pain, it can be unremitting. In contrast, vomiting occurs in episodes, albeit sometimes spread over many days. Further, the adaptive function of nausea (learning to avoid foods that caused its induction on a previous encounter) becomes a liability when it leads to avoidance and refusal of potentially curative therapy in the case of some anti-cancer chemotherapy (Maceira et al., 2012).

This review focuses on the identification of anti-emetic drugs for therapeutic use in humans. Not discussed are important veterinary applications, particularly in oncology (Kenward et al., 2017).

## Brief introduction to mechanisms

**Figure 5:**
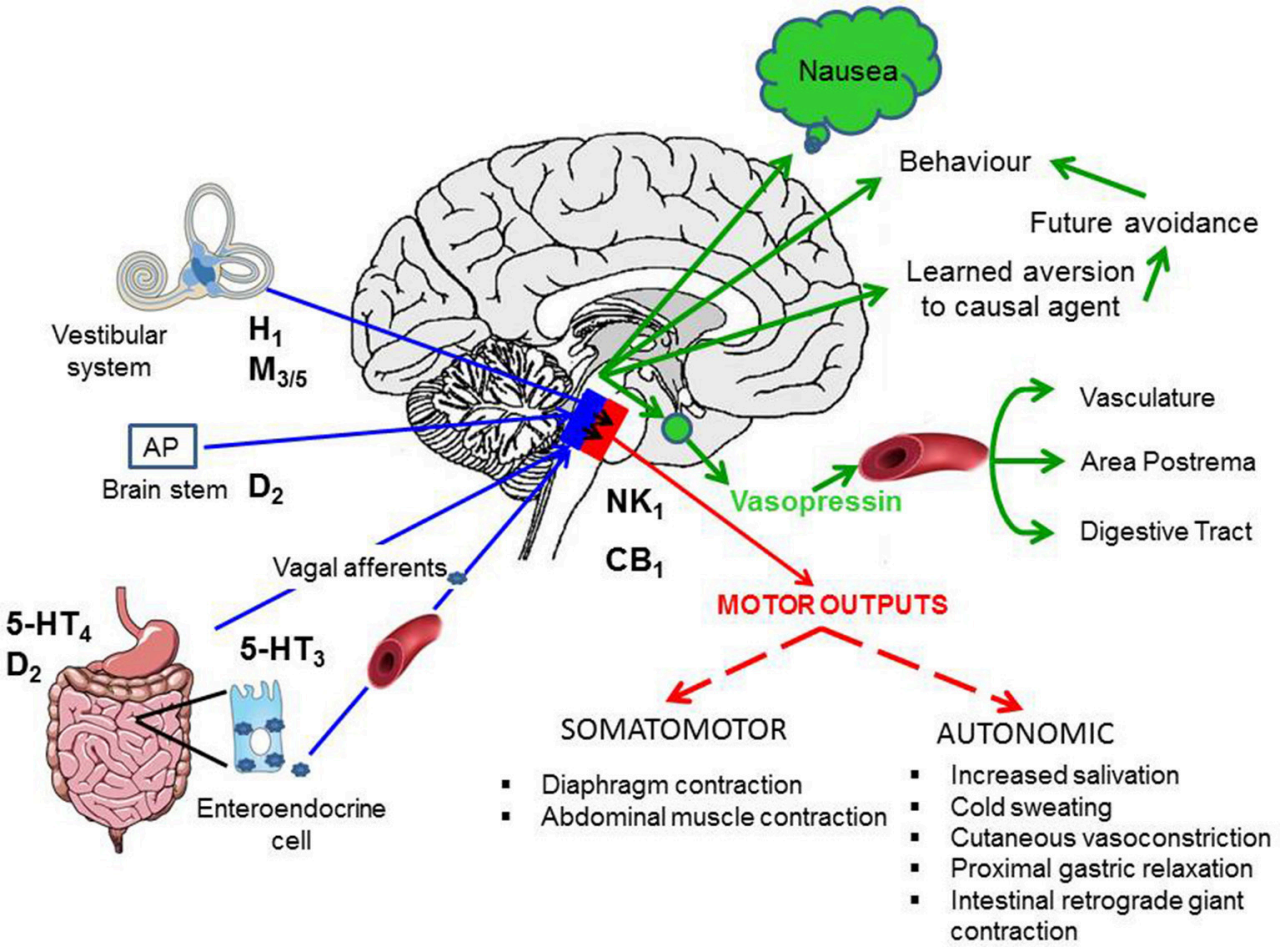
Summary of the pathways responsible for the induction of nausea and vomiting (blue arrows), the integrative regions in the brain stem (blue box indicates dorsal brain stem and nucleus tractus solitarius in particular) and the output pathways for nausea (green) and the motor outputs for vomiting (red box indicates the pathways in the ventral brain stem). See text for details of pathways. CB_1_, cannabinoid_1_ receptor; D_2_, dopamine_2_ receptor; H_1_, histamine_1_ receptor; M_3/5_, muscarinic_3/5_ acetylcholine receptor; 5-HT_3_-5-hydroxytryptamine_3_ receptor; 5-HT_4_-5-hydroxytryptamine_4_ receptor; NK_1_, tachykinin neurokinin _1_ receptor. Adapted and modified from Stern et al. (2011).

The pathways involved both in the induction and the motor outputs of emesis are briefly described, so the sites of action of anti-emetics (discussed below) can be identified (Figure 5).

### Major pathways

#### Vestibular system

Although motion sickness can be induced by the vestibular system alone (Irwin 1881; the first person to use the term “motion sickness”), it more often involves conflicting or discordant signals from the vestibular and visual systems, possibly with involvement of proprioceptive inputs (Money, 1970; Reason, 1978; Oman, 2012; Lackner, 2014; Yates et al., 2014; Golding and Gresty, 2015; Bertolini and Strauman, 2016).

#### Area postrema (AP)

Located at the caudal extremity of the IVth ventricle, the area postrema is characterized by relatively permeable blood-brain and cerebrospinal fluid-brain barriers. Lesion studies primarily in the 1950s and 1960s (e.g., Wang and Borison, 1952; for review see Borison, 1989) implicated this region in the emetic response to a diverse range of substances in the blood and led to its description as the “chemoreceptor trigger zone” (CTZ) for emesis. A widespread view then developed that agents in the circulation could *only* induce emesis via the AP, resulting in this region becoming a focal point for therapeutic intervention (see Domperidone, below) and distracting attention from the involvement of other pathways activated by systemic agents. Nevertheless, the importance of the AP is exemplified by its role in the induction of emesis by a number of endogenous circulating agents (e.g., adrenaline, glucagon-like peptide-1, cholecystokinin) as well as by drugs (e.g., apomorphine, digoxin, morphine; see Stern et al., 2011). The reliable activation of emesis by apomorphine via the AP led to its widespread use as a test stimulus for investigating potential anti-emetic agents but over-simplistic interpretation of the blockade of apomorphine-induced emesis by candidate drugs may have led to erroneous conclusions as illustrated by a quotation from Borison and McCarthy (1983, p. 16): “A misconception of the emetic mechanism that has led to false critical expectations is the idea that experimental drug antagonism of apomorphine-induced vomiting is equivalent to general inactivation of the chemoreceptor trigger zone.”

#### Abdominal vagal afferents

Projecting from the stomach and small intestine, vagal afferent neurons send information to the brain stem about the mechanical activity of the muscle and the chemical nature of the luminal environment. This includes the effects of distension, particularly of the gastric antrum and duodenum, which can induce nausea and vomiting but paradoxically, gastric motor quiescence is also associated with nausea (Sanger et al., 2013). Increasing evidence also points toward dysrhythmic gastric movements in certain conditions associated with nausea (e.g., gastroparesis) thought to be detected by vagal mechanoreceptors and signaled to the brainstem (Stern et al., 2011). In addition, the mucosal chemoreceptive vagal afferents are implicated in emesis caused by ingested luminal toxins and irritants. In this setting, the detection of substances in the lumen is via enteroendocrine cells within the mucosa, which release neuroactive substances (e.g., 5-HT, cholecystokinin) locally to activate receptors on the vagal afferents terminating in close proximity. Based upon direct and circumstantial evidence, Andrews et al. (1988) proposed that the enteroendocrine cells and the vagal afferents were involved in the acute emetic response to anti-cancer chemotherapeutic agents (e.g., cisplatin, cyclophosphamide) and abdominal radiation by the release of 5-HT (and other substances; see below) from the cells to act at 5-HT_3_ receptors on the vagal afferent terminals (see Andrews and Rudd, 2016 for review).

### Motor outputs

#### Vomiting

Vomiting is a reflex motor event coordinated in the brainstem. Classically, the term “vomiting center” described the brainstem locus from which vomiting could be induced when stimulated and was viewed as a conceptual target for anti-emetic drugs (Wang and Borison, 1950). Although “vomiting center” is a useful concept and is still used in text books (e.g., Rang and Dale's Pharmacology; Ritter et al., 2016), as the network of brainstem nuclei [e.g., nucleus tractus solitarius (NTS), dorsal motor vagal nucleus, Bötzinger complex] responsible for the genesis and coordination of the retching and vomiting motor pattern have been identified (Hornby 2001), such “black box” descriptions of networks may become redundant.

Key events in vomiting are: (a) Relaxation of the proximal stomach via reciprocal changes in activity of vagal inhibitory and excitatory neurons, together with a retrograde giant contraction (RGC) beginning in the lower small intestine and progressing to the stomach under vagal control (Lang, 2016). These changes confine potentially-contaminated gastric content to the stomach (the only place from which ejection by vomiting is possible) and the RGC returns already-emptied contents to the stomach. Retching only begins once the RGC reaches the stomach; (b) Contraction of the hiatal region of the diaphragm and inhibition of the crural diaphragm surrounding the lower esophagus by the phrenic nerve, and contraction of the abdominal muscles by the spinal motor neurons. It is these motor events which in terrestrial mammals provide the propulsive force for oral ejection of gastric contents (see Stern et al., 2011; Koch and Hasler, 2017).

#### Nausea

Compared with vomiting, nausea is poorly understood and difficult to define operationally (Stern et al., 2011; Balaban and Yates, 2017). There are, for example, fewer than 10 published human brain imaging studies investigating brain activity during nausea and all but one (Miller et al., 1996) used illusory self-motion as the stimulus. These studies implicate the anterior cingulate cortex (“visceromotor cortex”), inferior frontal gyrus, insular cortex and amygdala (Napadow et al., 2012; Farmer et al., 2015; Sclocco et al., 2016). In some brain areas (e.g., posterior cingulate cortex) the activity showed a negative correlation with nausea (Farmer et al., 2015). However, it must be emphasized that we do not yet know which regions are associated with the genesis of nausea and which are associated with the emotional and stressful aspects of the sensation and hence, are implicated in the associated autonomic changes characterized by increased sympathetic outflow. For a detailed review of the central pathways implicated in nausea, see Stern et al. (2011) and Koch and Hasler (2017).

Healthy volunteers and patients reporting nausea also have a number of physiological changes often referred to as “prodromata of vomiting.” The main ones are cold sweating (forehead) and pale skin pallor due to regional cutaneous vasoconstriction, tachycardia and increased heart rate variability, elevated plasma vasopressin (but not oxytocin) concentration indicative of hypothalamic-posterior pituitary involvement, and inhibition of gastric motility (see Stern et al., 2011, and Koch and Hasler, 2017).

The relatively poor temporal resolution of studies which have attempted to correlate physiological changes with the subject's reporting of nausea means that for elevated plasma vasopressin, gastric dysrhythmia and delayed gastric emptying, there is debate about the extent to which each contributes to the genesis of the sensation of nausea or are simply a component of the physiological response to activation of the emetic pathways (Stern et al., 2011; Andrews and Sanger, 2014). Resolving this “cause-consequence” conundrum is important for identifying which patient groups require therapeutic approaches that are directed centrally or peripherally.

Nausea is recognized as poorly treated in comparison to vomiting (Andrews and Sanger, 2014) and has been described as a “neglected symptom” during treatment of cancer patients (e.g., Foubert and Vaessen, 2005; Greaves et al., 2009; Jones et al., 2011). It is also one of the defining symptoms in the common, poorly-treated conditions of gastroparesis, functional dyspepsia and chronic unexplained nausea and vomiting (Sanger and Pasricha, 2017). However, such prevalence does not seem to have stimulated research to improve our understanding of the pathways involved in the etiology of nausea.

## Early serendipidous discoveries of anti-emetic drugs

The original drive to identify anti-emetic drugs most likely originated with the desire to block sea-sickness, with references to treatments in Classical Greek and Roman literature (Huppert et al., 2016) and more recently, Shakespeare (Cymbeline III, iv, 186; Kail, 1986). These and later attempts to block nausea and vomiting prior to and during World War II (WWII, 1939–1945) were largely based on traditional, historic and unproven remedies for sea-sickness, with more than 40 treatments identified based on publications in the Lancet between 1828 and 1928 (Reason and Brand, 1975). The only substances recognized in antiquity and pre-WWII and shown subsequently to have efficacy, are atropine and hyoscine (see below). This required development of methodologies for objective assessment of sea-sickness at sea and methods for induction of motion sickness in controlled laboratory conditions in humans and animals (McEachern et al., 1942; Noble, 1945; Babkin et al., 1946; Holling, 1947; Brand and Perry, 1966). The drug trials methodology developed by the United Kingdom military and the Medical Research Council became a model for drug trials in other areas.

By 1976, a series of largely serendipitous developments identified four categories of anti-emetic drug (Gibbs 1976): (i) Anticholinergic drugs (later shown to antagonize muscarinic M_3_ and M_5_ receptors); (ii) Antihistamines (later shown to act predominantly as antagonists at the histamine H_1_ receptor but also at muscarinic receptors); (iii) Derivatives of phenothiazine (shown to act as dopamine D_2_ receptor antagonists but also with effects at other receptors); (iv) Metoclopramide, a drug derived from the local anesthetic procainamide (initially described as a D_2_ receptor antagonist before other activities were discovered some year's later; see below).

The early discoveries were made by testing in humans to confirm anecdotal reports (e.g., the anti-cholinergic hyoscine) or after rapid transition of a newly discovered molecule into the clinic, when anti-emetic activity was unintentionally discovered (e.g., antihistamines). Thereafter, animal studies began to appear more frequently, beginning with their use in the discovery of anti-emetic activity during routine screening for general activity (the phenothiazines) and then to characterize the actions of other dopamine_2_ (D_2_) receptor antagonists from chemical programmes initially directed at controlling psychiatric disorders.

The pharmacology (receptor affinities/potencies) and structures of the major anti-emetic drugs discussed in the sections below are summarized in Table 2.

**Table 2:** Structures, receptor affinities, and actions of anti-emetic drugs.

	**D_1_**	**D_2_**	**D_3_**	**D_4_**	**α_1_**	**α_2_**	**H_1_**	**M**	**5-HT_2_*A***	**5-HT_3_A**	**Other**
**Muscarinic Receptor Antagonist**											
**Scopolamine** 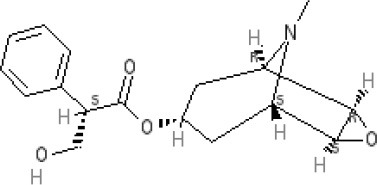		**K*_*i*_ >10,000 nM (rat)^13^*					**K*_*i*_ >10,000 nM (rat)^13^*	M_1_ **9.0** M_2_ **8.7** M_3_ **9.4** M_4_ **9.5** antagonist			
**Histamine H**_1_ **Receptor Antagonists**											
**Diphenhydramine** 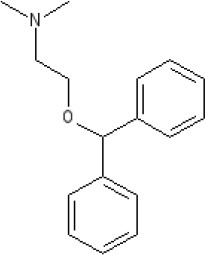		**K*_*i*_ >10,000 nM (rat)*^13^					**7.9** antagonist	*pA_2_ 7.1 antagonist (rat)^7^*			
**Promethazine** 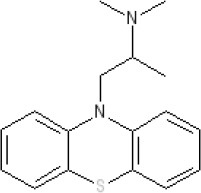		**K*_*i*_ 240 nM (rat)^13^*					**9.6** antagonist	**K*_*i*_ 21 nM (rat)^13^*			
**Cyclizine** 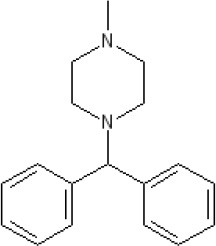							**8.4** antagonist				*Pre-ganglionic cholinergic inhibition (animals)^15^*
**Cinnarizine** 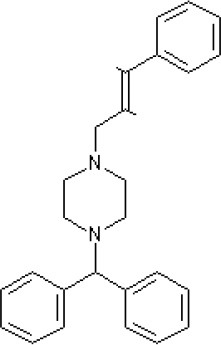							*antagonist^16^*	*antagonist^16^*			*Blocks L-type and T-type calcium channels^16^*
**Phenothiazines**											
**Prochlorperazine** 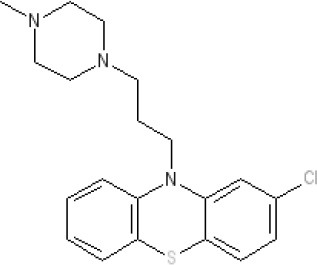	7.1 antagonist	**8.4** antagonist	**8.4** antagonist	6.1 antagonist	**K*_*i*_ 200 nM (rat)^14^*		*p1C_50_ 6.7 inverse agonist^6^ 8.2^8^*	**K*_*i*_ 2,100 nM (rat)^13^*	***8.2**^3^*	*Inactive (rat)^10^*	
**Chlorpromazine** 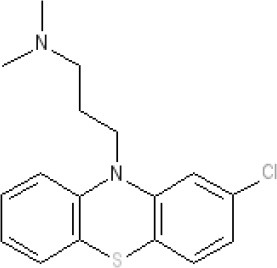	7.1 antagonist	7.0–7.6 antagonist	7.2–7.5 antagonist	7.8 antagonist	α_1*A*_ **K*_*i*_ 0.28 nM^12^*	α_2A_ 5.9–6.6 α_2B_ 7.2–8.3 α_2C_ 6.9–7.4 antagonist at each	**8.2** antagonist	**K*_*i*_ 47 M_3_ (rat)^12^*	**8.1** inverse agonist	*Inactive (rat)^10^*	D_5_ 6.9 antagonist 5-HT_1A_ 6.2 antagonist 5-HT_2C_ 7.6–8.2 antagonist 5-HT_6_ 7.7–7.8 inverse agonist 5-HT_7_ 7.6 inverse agonist
**Fluphenazine** 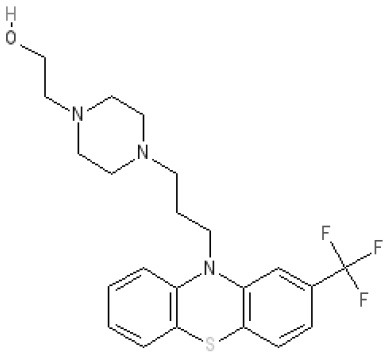	7.7 antagonist	**8.8** antagonist			**K*_*i*_ 8.1 nM (rat)^14^*		7.7 antagonist	**K*_*i*_ 340nM (rat)^13^*	7.5 antagonist	*Inactive (rat)^10^*	D_5_ 7.9 antagonist 5-HT_7_ 7.9 inverse agonist 5-HT_6_ 7.3–7.4 inverse agonist
**Levomepromazine** 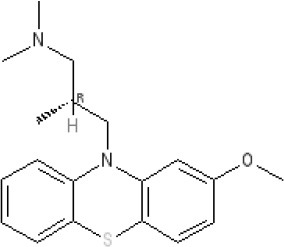	**K*_*i*_ 54.3^1^*	**K*_*i*_**8.6**^1^*	**K*_*i*_**8.3**^1^*				*K_*d*_ 0.58nM antagonist^2^*				
**Mirtazapine** 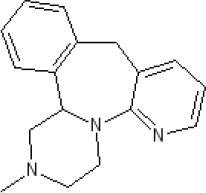					α_2A_ **7.7** antagonist	α_2C_ **7.7** antagonist	*p1C_50_ 9.6 inverse agonist^6^**8.98***		**7.2** antagonist		5-HT_2C_ **7.4** antagonist
**Metoclopramide**											
**Metoclopramide** 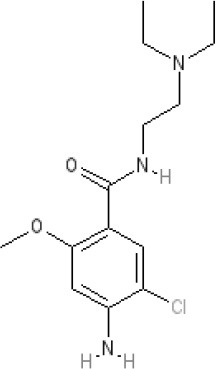		**7.5** antagonist (mouse)			**K*_*i*_ >10,000 nM (rat)*		**K*_*i*_ 1,100 nM (rat)^13^*	**K*_*i*_ >10,000 nM (rat)^13^*		5-HT_3_A **6.0–6.4** antagonist 5-HT_3_AB **5.7** antagonist	5-HT_4_ **6** agonist (mouse)
**Butyrophenones**											
**Haloperidol** 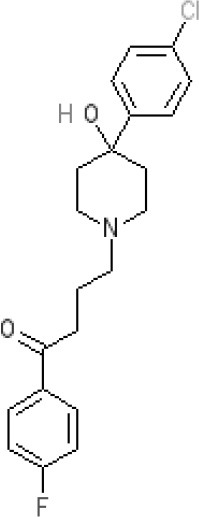	7.6–8.2 antagonist	**7.4–8.8** antagonist	**7.5–8.6** antagonist	**8.7–8.8** antagonist	**K*_*i*_ 46 nM^5^*	**K*_*i*_ 360 nM^5^*	5.7–6.1 antagonist	**K*_*i*_ >1,000 nM at M_1_, M_2_, M_3_ (rat)^5^*	6.7–7.3 antagonist	**K*_*i*_ >1,000 nM^5^*	5-HT_1D_ 6.6 antagonist 5-HT_7_ 6.3–6.6 antagonist 5-HT_2B_ 5.8–6.4 antagonist 5-HT_1A_ 5.7–5.8 antagonist
**Droperidol** 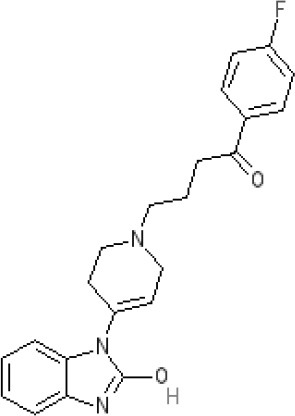		**K*_*i*_ 3 nM (rat)^11^*			**K*_*i*_ 1.4 nM (rat)^11^*		**K*_*i*_ 2,500 nM (rat)^11^*		**K*_*i*_ 4.6 nM (rat)^11^*	*Inactive (rat)^10^*	
**Domperidone** 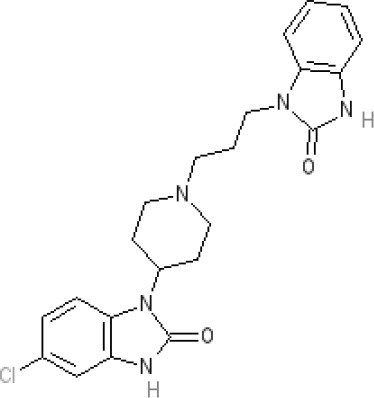		**7.9–8.4** antagonist	**7.1–7.6** antagonist	**K*_*i*_ 30.4 nM^4^*	**K*_*i*_ α_1*A*_, 71; α_1*B*_, 530; α_1*D*_, 710 nM^9^*					*Inactive (rat)^10^*	
**Second generation anti-psychotics**											
**Olanzapine** 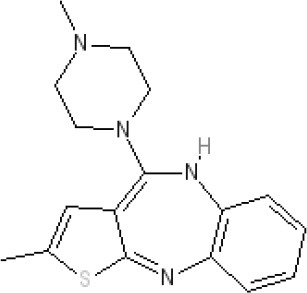	**K*_*i*_ 31 nM (rat)^5^*	**8.7** antagonist		**K*_*i*_ 27 nM (rat)^5^*	α_1*A*_ **K*_*i*_ 115 nM^12^*	*α_2*A*_ Ki 314; α_2*B*_ 81.6; α_2*C*_ 28.8 nM^12^*	**8.7–9.2** antagonist	**K*_*i*_ 105 nM at ${\text{M}}_{3}^{12}$*	**8.6–8.9** antagonist	**K*_*i*_ 57 nM (rat)^5^*	5-HT_2C_ **8.1–8.4** inverse agonist 5-HT_6_ **8** inverse agonist 5-HT_7_ 6.5 antagonist
**5-HT**_3_ **Receptor Antagonists**											
**Granisetron** 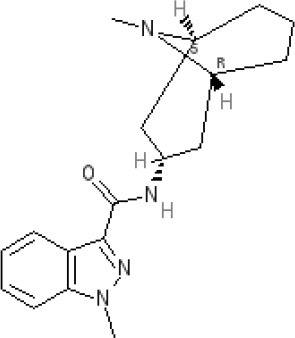		*Inactive (>10,000 nM) (rat)^10^*								5-HT_3_A ~**8.6–8.8** antagonist	
**Ondansetron** 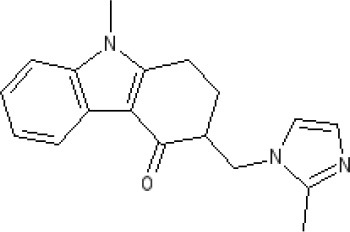										5-HT_3_A ~**7.8–8.3** 5-HT_3_AB **7.8** antagonist	
**Tropisetron** 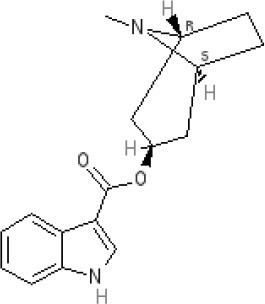		*Inactive (>10,000 nM) (rat)^10^*								5-HT_3_A **8.5–8.8** antagonist	5-HT_4_ 6.3–7.1 antagonist
**Palonsetron** 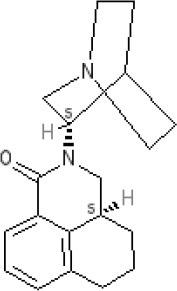										5-HT_3_A **10.5** antagonist	
**NK**_1_ **receptor antagonists**											
**Aprepitant** 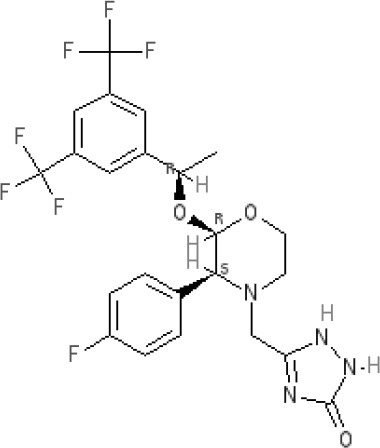											NK_1_ **10.1** antagonist

*Data are from http://www.guidetopharmacology.org/ and are given as pK_i_-values for the human receptors (usually recombinant); primary affinities are in bold. Additional information on drug affinity for human receptors (unless otherwise specified) is provided in italics together with references, listed below. Little information is known for cyclizine or for cinnarizine, particularly in terms of their interactions with human receptors and mechanisms. Clarke and Dundee (1971) describe significant sedation/drowsiness associated with promethazine, thiethylperazine, cyclizine and hyoscine. ^1^Srivastava et al., 2009; ^2^Kanba and Richelson, 1984; ^3^Meltzer et al., 1989; ^4^Moreland et al., 2004; ^5^Bymaster et al., 1996; ^6^Bakker et al., 2007; ^7^Liu et al., 2006; ^8^Appl et al., 2012; ^9^Keiser et al., 2009; ^10^Hamik and Peroutka, 1989; ^11^Peroutka and Synder, 1980; ^12^Kroeze et al., 2003; ^13^Peroutka and Snyder, 1982; ^14^Ison and Peroutka, 1986; ^15^Norton et al., 1954; ^16^https://www.drugbank.ca/drugs/DB00568#BE0000442*.

### Hyoscine and scopolamine

The alkaloids hyoscyamine and hyoscine (also known as scopolamine) are found in different plants from the family *Solanaceae* (e.g., hyoscyamine in the deadly nightshade *Atropa belladonna* and hyoscine from henbane, *Hysoscyamus niger* (Henry, 1939). The toxic and medicinal properties of this plant family have been known since antiquity (see Thearle and Pearn, 1982). Extraction of the naturally-occurring levorotatory isomer of hyoscyamine leads to formation of the racemic mixture known as atropine (Sneader, 2005).

Although this class of drug was suggested to be effective against seasickness as long ago as 1881 (Irwin, 1881) it was not until WWII that structured trials investigated the activity of potential anti-emetic medications including hyoscine, atropine, the different enantiomers of hyoscyamine, phenobarbitone, sodium hydantoinate, chloretone, syntropan, hexobarbitone, and methidrine (Reason and Brand, 1975). The trials occurred using mine sweepers sent to sea in rough weather and positive responders were those who did not experience nausea and/or vomiting. The results, for the first time, demonstrated the preventative efficacy of hyoscine in particular and also atropine and the l-isomer of hyoscyamine (Holling et al., 1944; Holling, 1947). These studies were rapidly followed by demonstration of the anti-emetic efficacy of hyoscine among soldiers in assault craft during tropical conditions (Hill and Guest, 1945). Today, drugs such as scopolamine are widely available for the treatment of all causes of motion sickness, manufactured by different companies in oral formulations and in more convenient formulations for anyone already experiencing nausea, such as transdermal patches and nasal sprays (Spinks and Wasiak, 2011; Golding and Gresty, 2015). Following identification of the different human muscarinic receptor subtypes (Huang et al., 2001) these drugs have been shown to act most notably at the M_3_ and M_5_ receptors which mediate cholinergic activity within the vestibular input to the vestibular nuclei and probably also within brainstem pathways integrating vomiting such as the NTS (Golding and Stott, 1997; Soto and Vega, 2010)

### Antihistamines

The discovery of the “antihistamines” (the term histamine receptor antagonist was not introduced until 1966; Ash and Schild, 1966) was initiated by academic curiosity in 1937 (at the time compounds were known to block the actions of adrenaline and acetylcholine, so why not histamine?) and then rapidly further developed by the pharmaceutical industry. Initial success was achieved by Rhône-Poulenc Laboratories (Tables 1, 2) screening “libraries” of compounds previously synthesized during a search for therapeutic alternatives to the anti-malaria drug quinine (from compounds traditionally used in the dying industry but known to exert anti-septic, anti-helminthic and anti-malarial activity), the supply of which was hindered by blockades imposed on Germany during WWI and then in WWII by Japanese expansion into South-East Asia (López-Muñoz et al., 2005). The first antihistamine to treat anaphylaxis and allergic reactions was phenbenzamine (also known as antergan), introduced into the clinic by Rhône-Poulenc in 1942. This was followed by diphenhydramine, chlorpheniramine, brompheniramine, promethazine and cyclizine (Emanuel, 1999; Sneader, 2005; Church and Church, 2013). Notably, H_1_ receptor antagonism also supresses a number of different pathways within the brain, including those involved in arousal, leading to drowsiness, somnolence and sedation. As a counter-measure, dimenhydrinate (Dramamine) was introduced by G.D. Searle & Co, consisting of diphenhydramine with 8-chlorotheophylline (a mild stimulant and derivative of theophylline). Later, in the 1980s, other compounds were identified with poor ability to cross the blood brain barrier, the so-called “second generation” H_1_ receptor antagonists, which do not have anti-emetic activity (Slater et al., 1999; Simons and Simons, 2011).

**Figure 6:**
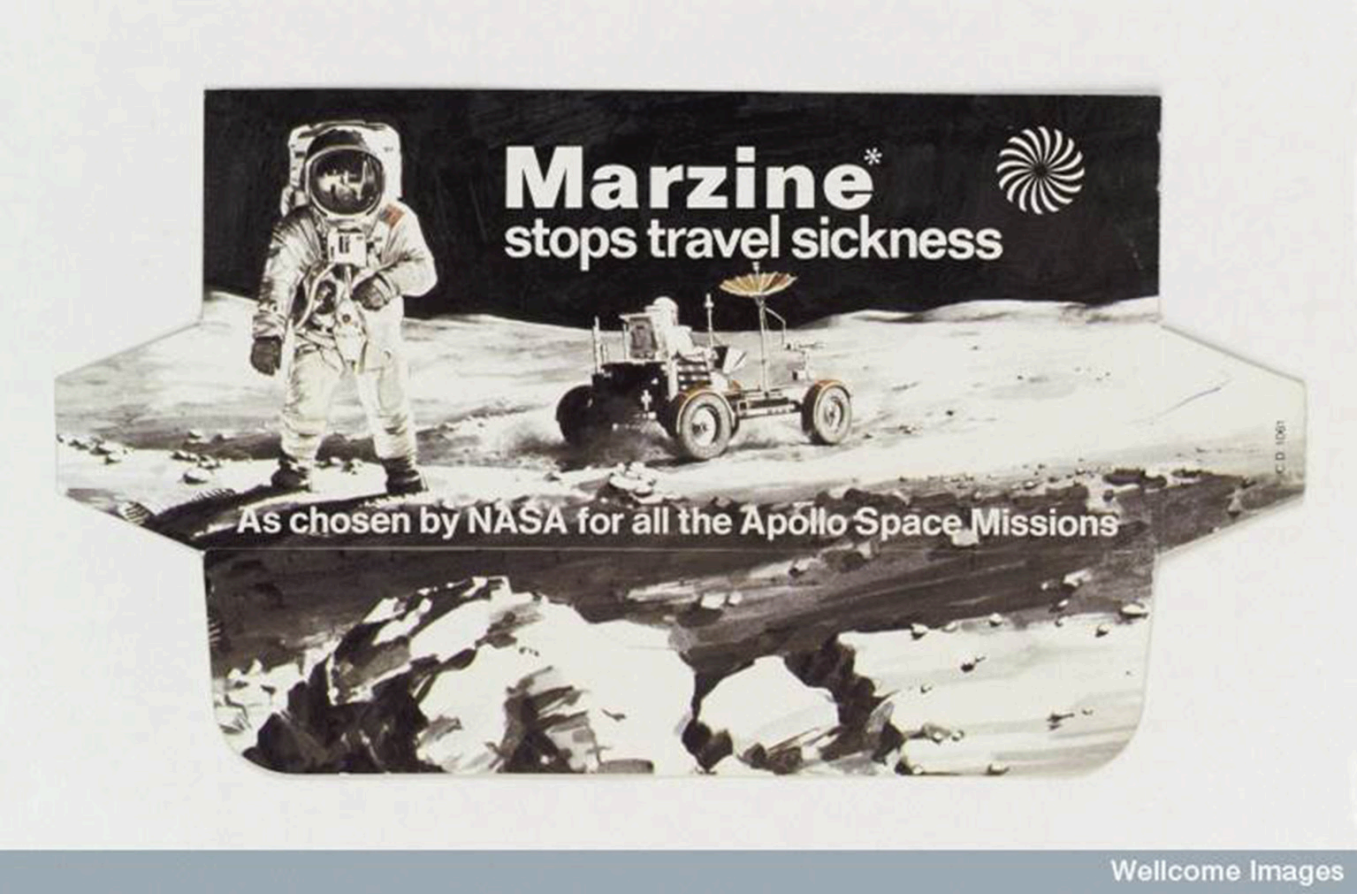
Photograph of the packaging for Marzine (cyclizine, developed in 1947) indicating its use by NASA during the Apollo moon missions. With permission: Wellcome collection, Wellcome Library (WF/M/PL/191), London, United Kingdom.

The discovery of antiemetic activity among the first generation antihistamine drugs was serendipitous. Dimenhydrinate (Dramamine) was undergoing evaluation in 1947 as a potential treatment of hay fever and urticaria. Among the patients receiving the drug was a pregnant woman who suffered from car sickness all her life. However, if she took dimenhydrinate a few minutes before boarding a tramcar she remained symptom-free; placebo was ineffective (Gay and Carliner, 1949). Next year (1948) G.D. Searle & Co conducted a trial in which dimenhydrinate or placebo was given for 10 days or as a successful rescue therapy to 485 male USA troops crossing the Atlantic during “a rough passage” in the General Ballou, a converted freight ship (Gay and Carliner, 1949). In 1949 diphenhydramine itself (Benadryl) was shown to alleviate nausea and vomiting induced by streptomycin in four patients with pulmonary tuberculosis (Bignall and Crofton, 1949). These trials established the use of antihistaminic drugs as treatments of motion sickness and indicated that they may also be effective against emesis induced by other challenges. Cyclizine, developed in 1947 by Burroughs Wellcome, was shown to prevent sea- and air-sickness in 1952–1953 (see Norton et al., 1954 for references and data on the autonomic pharmacology of cyclizine) and has the notable history of being taken to the moon as a treatment for space sickness (Figure 6).

The first generation “antihistamines” (used to treat various allergic conditions; Simons and Simons, 2011) were effective against motion sickness, nausea and vomiting caused by labyrinthine disturbances (e.g., labyrinthitis and fenestration operations; Wang, 1965) and were investigated as anti-emetics in a number of other clinical settings (e.g., PONV, see Palazzo and Strunin, 1984; pregnancy, see Fairweather, 1978 and also Bhargava and Dixit, 1968, for pre-clinical studies). As anti-emetic drugs they are effective because they block H_1_ receptors in the vestibular system and also in the brainstem integrative circuitry (“vomiting center”) (Takatani et al., 1983; Soto and Vega, 2010). However, for some compounds, additional anti-emetic activity is thought to be due to their additional ability to antagonize at muscarinic receptors, perhaps not surprising, given the origin of the early compounds from a chemical template used to identify “adrenergic” and “cholinergic” antagonists (Liu et al., 2006). For example, in addition to antagonizing at the human H_1_ receptor (Ki 12.6 nM), diphenhydramine also inhibits M_2_ receptors (estimated Ki 80 nM) and displaces QNB binding in the cerebral cortex (Ki 280 nM; Kubo et al., 1987; Booth et al., 2002; Liu et al., 2006). Similarly, cyclizine and promethazine antagonize at the human H_1_ receptor (respectively, Ki-values of 4.44 and 0.24 nM; Chazot et al., 2017) and appear to have an ability to inhibit the functions of acetylcholine (Norton et al., 1954). These drugs had no ability to prevent the vomiting initiated by apomorphine, a D_2_ receptor agonist acting on the AP (see Carpenter et al., 1983 and also Borison and Wang 1953 and Borison, 1989 for review of evidence on the effect of area postrema ablation on the emetic response to apomorphine).

Histamine_1_ receptor antagonists, in addition to illustrating how the pharmacological profile of compounds may change from that originally described, are also examples of a more fundamental shift in pharmacological characterization. Although the agents described above such as diphenhydramine are commonly referred to as H_1_ receptor “antagonists,” modern pharmacology now classifies them as “inverse agonists” (Bakker et al., 2007; Simons and Simons, 2011) but the implications of this for understanding both the mechanisms of emesis and the anti-emetic effects of different H_1_ “antagonists” have not yet been considered (Tu et al., 2017).

### Phenothiazines

The term “phenothiazines” refers to compounds with a nucleus of two benzene rings linked by a sulfur and a nitrogen atom to form a heterocyclic 3-ring compound, with phenothiazine itself first synthesized in 1883 (see Wang, 1965 for review). Chlorpromazine (Thorazine) was discovered from the observation that certain anti-histamines, in addition to prolonging sleep induced by barbiturates, also reduced the “shock” of surgery when given during anesthesia, somehow depressing the nervous system to leave patients relatively calm and relaxed during recovery. Re-examination of the antihistamines to optimize the “anti-shock” property (e.g., by testing for an increase in time required for trained rats to climb a vertical rope for food) led to synthesis of chlorpromazine (or 4560-R.P) in 1946 (Sneader, 2005). This compound had low antihistamine activity but blocked the effect of adrenaline on blood pressure and in research within SmithKline and French, inhibited conditioned reflexes in rats. The compound also prevented emesis evoked by apomorphine, acting on the AP in dogs (Glaviano and Wang, 1955). Apomorphine is primarily considered to be a D_2_ receptor agonist but it is now more accurately defined as a potent agonist at the D_2_ receptor subfamily (D_2_, D_3_, D_4_) and D_5_ receptors, with additional affinity for alpha_1_- and alpha_2_-adrenoceptors, 5-HT_1A_ and 5-HT_2_ (5-HT_2A_, 5-HT_2B_, 5-HT_2C_) receptors (Millan et al., 2002).

The pharmacological data on chlorpromazine, generated by Rhône-Poulenc in 1951, were published (Courvoisier et al., 1953) after the first clinical evaluation for treatment of “surgical shock” in 1952. The commercial name for chlorpromazine (Largactil) reflected its broad spectrum of activity (“large” = broad or wide, “acti” = activity) (López-Muñoz et al., 2005). Later, Carlsson (Nobel Prize Winner) and Lindqvist (1963) showed that chlorpromazine binds to postsynaptic dopamine receptors, launching the “dopamine hypothesis of schizophrenia” (in which symptoms could be treated by blocking dopamine receptors in post-synaptic neurons; Snyder et al., 1974) and revolutionizing treatment of psychiatric disorders.

Chlorpromazine was originally used to treat “neurosis” (sedation in psychiatric patients) and as pre-anesthetic medication, inhibiting nausea and vomiting, “shock” and augmenting the effects of anesthetics (Moyer et al., 1955). The anti-emetic activity of chlorpromazine was evaluated in more detail by Boyd et al. (1953, 1954) using dogs and apomorphine. This work was initiated after Prof. R. Paul (Faculté libre des Sciences d'Angers) visited his laboratory in November 1951, during which he described experiments at Rhone-Poulenc, as yet unpublished, showing 4560-R.P potentiating the action of sedatives and inhibiting apomorphine-induced vomiting in dogs. Prof. Paul arranged to have some sent to his laboratory, so its anti-emetic activity could be compared with promethazine, a structurally-related compound the authors had previously reported to have limited anti-emetic activity. The results clearly demonstrated the ability of chlorpromazine to prevent apomorphine-induced emesis in dogs. Contemporaneously, Brand et al. (1954) reported similar findings in dogs, using apomorphine, morphine and ergot as the emetic stimuli, but failed to prevent emesis evoked by copper sulfate or inhibit the response to any emetic stimulus in cats. In addition, the structurally related antihistamine, promethazine (Phenergan) had no ability to inhibit apomorphine-induced emesis. These data were consistent with Schmidt et al. (1953) who used dimenhydrinate and diphenhydramine. Later, a more detailed comparison using a number of phenothiazines (chloropromazine, promazine, trifluoperazine, levomepromazine, prochlorpromazine), trimethoxybenzamide, antihistamines (perphenazine, thiethylperazine, dimenhydrate, cyclizine), and hyoscine (Wyant, 1962) confirmed and extended these observations in dogs, demonstrating the ability of the phenothiazines and trimethoxybenzamide to prevent apomorphine-induce vomiting but to have lower activity against emesis evoked by intra-gastric copper sulfate, whereas the reverse was demonstrated by the antihistamines and by atropine.

These data were interpreted by reference to a series of experiments into the mechanisms and pharmacology of vomiting, reviewed by Borison and Wang (1953). The authors determined that vomiting induced by apomorphine (primarily a D_2_ receptor agonist; see earlier) was caused by direct stimulation of the AP, considered the site at which emetic substances in the blood could induce emesis. Thus, chlorpromazine and the other phenothiazine derivatives acted primarily by blocking dopamine receptors (the term D_2_ receptor was introduced by Kebabian and Calne, 1979) and although previously suggested, it was not until 1981 that the presence of D_2_ receptors within the AP of dogs was confirmed (Stefanini and Clement-Cormier, 1981). The drugs also exerted some general sedative effects, but failed to prevent emesis induced by intra-gastric copper sulfate via visceral afferent activation.

Chlorpromazine was first evaluated as an anti-emetic in humans by cautious administration to patients with terminal cancer or uremia and then, following success, it was given to patients with a range of disorders, including labyrinthitis, psychological vomiting and pregnancy sickness, in addition to patients suffering from vomiting induced by a variety of drugs (Friend and Cummins, 1953, 1954).

Wampler (1983) provides the structures of the different phenothiazines and discusses their relative efficacies and adverse events. In summary, there is little evidence for differences in anti-emetic activity but differences in “anti-adrenergic,” “anti-histaminic,” and “anti-serotonin” activities confer variations in side-effects of sedation and hypotension. The strong “anti-adrenergic” activity of chlorpromazine, for example, was associated with hypotensive side-effects. Today, chlorpromazine has been shown to have approximately similar affinity for human H_1_, α-adrenoceptor_2B_, D_2_, D_3_ and 5-HT_2C_ receptors (acting as an antagonist) and for 5-HT_2A_ and D_5_ receptors, acting as an inverse agonist^4^ Examples of piperazine side-chain phenothiazines that have potent antiemetic activity include perphenazine, prochlorperazine and thiethylperazine maleate. These drugs (particularly prochlorperazine) were rapidly adopted for clinical use in a number of settings including anti-cancer chemotherapy, later becoming the comparator for newer agents (e.g., metoclopramide, cannabinoids; see Harris and Cantwell, 1986).

### Metoclopramide

This drug was identified by Laboratoires Delagrange in France in the mid-1950s, during a programme aimed at improving the properties of procainamide, a cardiac anti-arrhythmic and local anesthetic drug derived from procaine. Although some anti-emetic activity was known to exist within this class of molecule, chlorination of the benzene ring of procainamide (2-chloroprocainamide) significantly increased anti-emetic activity in dogs. However, more interesting was the absence of the sedative activity of the phenothiazine structures prompting an evaluation of related structures. In particular, methoxy-2-chloro-5-procainamide or metoclopramide, had negligible local anesthetic or cardiac anti-arrhythmic activity but an ability to inhibit emesis in dogs evoked by apomorphine and hydergine, in addition to copper sulfate (Justin-Besancon et al., 1964). Soon after, metoclopramide was found to stimulate gastric emptying, speed the rate of transit through the small intestine and reduce symptoms associated with various upper digestive tract disorders (Boisson and Albot, 1966; Robinson, 1973; Schulze-Delrieu, 1979; Gralla, 1983; Sanger and King, 1988). Between 1967 and 1971 several clinical trials evaluated the ability of metoclopramide to inhibit emesis, mostly in patients experiencing PONV, with perphenazine, trimethobenzamide, prochlorperazine and perphenazine as the comparators (Robinson, 1973). Delagrange undertook limited marketing of metoclopramide, also licensing to A. H. Robins (later acquired by American Home Products, which changed its name to Wyeth) for the USA markets, and with some initial skepticism over its wide range of potential clinical usage (Robinson, 1973), to Beecham Pharmaceuticals in the UK.

As dopamine receptors were characterized (Kebabian and Calne, 1979), metoclopramide was shown to be a D_2_ receptor antagonist, selective over the D_3_ receptor and the α_1_-adrenoceptor (Rosenfeld et al., 1982; Andrews and Sanger, 2014). The drug found widespread use as an anti-emetic (e.g., during post-operative care or for patients with gastritis, migraine, dysmenorrhea and drug- or treatment-induced forms of emesis including that caused by anesthesia, radiation and some anti-cancer chemotherapies) and as a stimulant of upper gut motility (e.g., patients with gastro-esophageal reflux disease, gastroparesis, and functional dyspepsia; Pinder et al., 1976; Harrington et al., 1983). Initially, both the anti-emetic and prokinetic activities were attributed to dopamine receptor antagonism (Table 3). Although a major drug (there are now many generic versions across the world), its limited central action as an anti-emetic is, nevertheless, illustrated by its relative ineffectiveness in motion sickness. Further, at conventional doses (20 mg × 3 orally), the drug showed little or no anti-emetic superiority over placebo or prochlorperazine, when evaluated against the highly emetogenic agent cisplatin (e.g., Moertel and Reitemeier 1969), a relatively new anti-cancer drug at the time.

**Table 3:** Changing understanding of the role of gastric motility in the genesis of nausea and vomiting: Influences on drug discovery.

	**CONCEPT: Gastric prokinetics help patients with delayed gastric emptying including functional dyspepsia/gastroparesis (multiple symptoms, including nausea, vomiting, early satiety)**	
**Mid-1950s** Metoclopramide synthesized^1^	**5-HT**_4_ **receptor agonists** Metoclopramide •5-HT_4_ agonist, D_2_ antagonist (later shown to be a 5-HT_3_ antagonist)^2^ •Used in GERD, functional dyspepsia, gastroparesis; the only prescribed drug for gastroparesis in the USA^3^	[-2pt]**CURRENT STATUS:** 1. Gastric Prokinetics (5-HT_4_ and motilin agonists) useful in patients requiring more rapid delivery of (for example) orally administered drugs to the intestine^17^ 2. No consistent correlation between symptoms (e.g., nausea, early satiety) and delayed gastric emptying^18^ 3. Gastric prokinetic and direct anti-emetic activity of metoclopramide confuses mechanism of therapeutic action 4. Role of erythromycin in the treatment of gastroparesis uncertain
Other substituted benzamides (eventually shown to be 5-HT_4_ receptor agonists)	Cisapride •5-HT_4_ agonist, poor D_2_ antagonist (later shown to have similar affinity for 5-HT_2A_, 5-HT_2B_, α_1_-adrenoceptors and low affinity for 5-HT_3_)^4, 5^ •5-HT_2A_ and 5-HT_2B_ also implicated in mechanisms of emesis^6^ •Reduced nausea in certain patients (now withdrawn)^7, 8^	
Explored	Others •Some animal data suggests ability to inhibit vomiting but non-selectivity of action makes it difficult to interpret^9^ •Gastroprokinetic activity may oppose ability of 5-HT_3_ antagonists to inhibit severe emesis in ferrets^10^	
**1989** Erythromycin proposed to act as a motilin receptor agonist^11^	**Motilin receptor agonists** Erythromycin •Antibiotic drug used at lower doses to treat patients with gastroparesis and delayed gastric emptying^12^ •Activates motilin receptors in enteric nervous system (prokinetic activity) and vagus^13, 14, 15^ •Low doses may have anti-emetic activity; high doses cause emesis^15^ •Limited by potential to exacerbate bacterial resistance, prolong cardiac QTc interval, and interact with cytochrome P450 CYP 3A4^12^ •The selective motilin agonist camicinal shown to promote gastric emptying and facilitate oral drug delivery in patients with Parkinson's disease^16^	
	**CONCEPT: Selective dopamine D**_2_ **antagonists are anti-emetic but also increase gastric emptying, making them additionally useful treatments of gastroparesis (as defined by delayed gastric emptying)**	
**1974** Domperidone synthesized^19^	Domperidone Increased gastric emptying in gastroparesis^20^ Alleviates symptoms of gastroparesis^21^ No effects on gastric emptying in healthy volunteers^22^ or in patients requiring video capsule delivery to the small intestine^23^ and no direct ability to influence contractility of human isolated stomach^24^ Low risk of cardiac QTc prolongation^25^ Registered for use in many countries but not in the USA^21^	**CURRENT STATUS:** 1. Domperidone still explored in treatment of gastroparesis^21^ 2. Selective 5-HT${}_{3}^{25}$ and NK_1_ antagonists^27^ have anti-emetic effects but do not increase gastric emptying although they may have benefits in patients with gastroparesis. 3. These data support a role for dopamine in regulation of gastric motility in addition to emesis during disease
	**CONCEPT: Ghrelin agonists increase gastric emptying, leading to exploration of their potential to treat gastroparesis, enhanced by ability to promote appetite/reduce emesis**	
**1999** Ghrelin discovered and sequenced^28^	•Increase gastric emptying in healthy volunteers and in patients with gastroparesis but may not be sustained with long-term dosing^29^ •No direct ability to influence contractility of human isolated stomach^30^ •Increases appetite and reduced nausea in patients, including gastroparesis^31^	**CURRENT STATUS:** 1. Ghrelin agonists remain of interest because they can reduce nausea and increase appetite^29^
	**CONCEPT: Dysrhythmic movements of the stomach cause nausea and/or are the result of nausea**	
**2017** Resurgence of interest in the relationships between gastric dysrhythmia, gastric emptying, nausea and vomiting and gastric pathology in patient sub-groups	Gastric Dysrhythmia •Association between nausea and dysrhythmia of gastric myoelectric activity characterized using electrogastrography in several groups of patients, strengthened by studies with dense recording arrays^32, 33^ Interstitial Cells of Cajal (ICC) •Responsible for electrical slow waves; damage associated with gastric dysrhythmia (initiation, propagation)^34^ •Hypothesis: Nausea caused by vagal afferents detecting gastric dysrhythmia and signaling to brain stem^32, 35^	**CURRENT STATUS:** •1. Exploratory research of ICCs as drug targets •2. Improved clinical classification of patient groups with delayed gastric emptying

*^1^Justin-Besancon and Laville, 1964; ^2^Sanger, 2009; ^3^Camilleri et al., 2013; ^4^Briejer et al., 1995; ^5^Smith et al., 2008; ^6^Johnston et al., 2014; ^7^Creytens, 1984; ^8^Bergeron and Blier, 1994; ^9^Sanger et al., 2013; ^10^Miner et al., 1987; ^11^Peeters et al., 1989; ^12^Sanger et al., 2013; ^13^Broad et al., 2012; ^14^Broad and Sanger, 2013; ^15^Javid et al., 2013; ^16^Marrinan et al., 2018; ^17^Sanger and Alpers, 2008; ^18^ Janssen et al., 2013; ^19^Champion et al., 1986; ^20^Ahmad et al., 2006; ^21^ Heckert and Parkman, 2018; ^22^Markey and Shafat, 2012; ^23^McFarlane et al., 2018; ^24^Sanger, 1985c; ^25^Ortiz et al., 2015; ^26^Midani and Parkman, 2016; ^27^Pasricha et al., 2016; ^28^Kojima et al., 1999; ^29^Sanger and Furness, 2016; ^30^Broad et al., 2014; ^31^Camilleri and Acosta, 2015; ^32^Koch, 2014; ^33^O'Grady et al., 2012; ^34^Angeli et al., 2015; ^35^Sanger and Pasricha, 2017*.

During the 1980s it was discovered that metoclopramide possessed an additional ability to stimulate gastric motility by activating 5-HT_4_ receptors and at higher concentrations than those required to antagonize at the D_2_ receptor, acting as a 5-HT_3_ receptor antagonist (Sanger, 2009; see below). The former provided the mechanism by which metoclopramide stimulated GI motility and the latter heralded the development of new anti-emetic treatments and a revolution in care of cancer patients. These developments occurred during a time when 5-HT receptor pharmacology was being redefined.

The classification of 5-HT receptors began in 1957 when using guinea-pig ileum as their model, Gaddum and Picarelli defined a 5-HT M receptor (neuronally-mediated muscle contractions, blocked by morphine and also by atropine, cocaine, and methadone, even after dibenzyline) and a 5-HT D receptor (non-neuronally-mediated smooth muscle contractions, blocked by dibenzyline and also by lysergic acid diethylamide, dihydroergotamine, and 5-benzyloxygramine, even after morphine; Gaddum and Picarelli, 1957). In 1986 the classification was updated and three receptors defined: 5-HT_2_ (5-HT D), 5-HT_3_ (5-HT M) and a tentative “5-HT_1−like_” receptor, with similarities to a heterogeneous group of 5-HT_1_ (high affinity) binding sites (Bradley et al., 1986). Today, seven different 5-HT receptors have been cloned and characterized, with subtypes for some of these. All are GPCRs except 5-HT_3_, a ligand-gated cation channel with potentially heterogeneous subunits (5-HT_3_A-E; Holbrook et al., 2009).

In the 1980s a growing understanding of the mechanisms of action of metoclopramide became a significant factor in the discovery of the 5-HT_4_ receptor. Firstly, it became clear that D_2_ receptor antagonism could not fully explain how metoclopramide increased GI motility; for example, the more selective D_2_ receptor antagonist domperidone did not mimic the ability of metoclopramide to facilitate cholinergic activity in human isolated stomach, thought to model the cholinergic-mediated gastric prokinetic activity of this drug (Sanger, 1985a). Thus, it was argued that metoclopramide acted on cholinergic nerves within the enteric nervous system (ENS), but not necessarily on other cholinergic neurons outside the ENS. Clearly, this activity in human isolated stomach was independent of brain function, consistent with the inability of vagotomy to prevent the prokinetic effects of metoclopramide (Jacoby and Brodie 1967). These and other experiments demonstrated that metoclopramide facilitated ongoing cholinergic activity, increasing the release of acetylcholine (ACh) rather than directly stimulating muscarinic receptors (Sanger, 2017). This activity was not due to antagonism at pre-junctional muscarinic receptors, was not blocked by antagonists at the adrenoceptors or D_2_ receptors, or by antagonists at various other receptors and mechanisms. Instead, relatively high concentrations of 5-HT mimicked the response and non-selective ligands for 5-HT receptors mimicked or blocked this action of metoclopramide (Sanger, 1985b,c, 1987a); the notable exception was the failure to mimic or inhibit with a 5-HT_3_ receptor antagonist, leading to the proposal that metoclopramide and related compounds such as renzapride, facilitated cholinergic activity within the ENS by activating a “myenteric 5-HT-like receptor” (Sanger, 1987a,b). This was quickly linked to a “non-classical” 5-HT receptor identified by Dumuis et al. (1998) in mouse embryo colliculi neurons and in guinea pig hippocampal membranes and later defined as the 5-HT_4_ receptor (Bockaert et al., 1992).

### Domperidone

**Figure 7:**
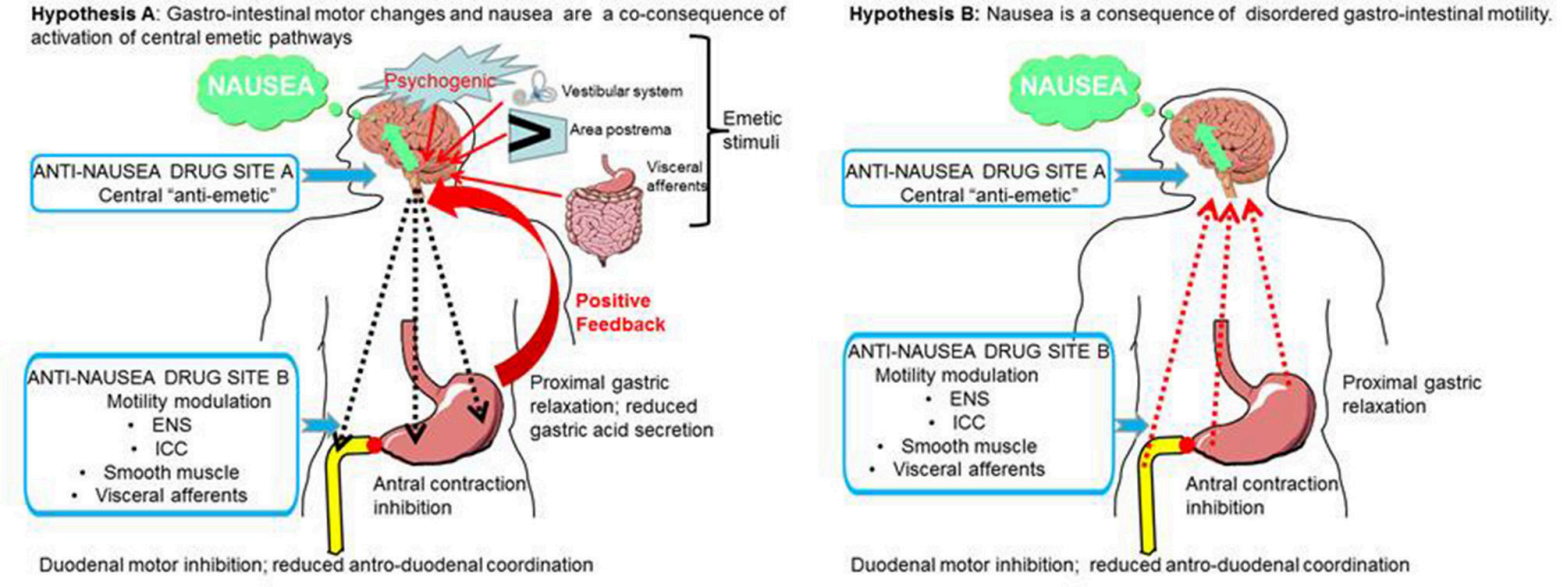
Two hypotheses for the relationship between disordered upper gastrointestinal tract motility and the sensation of nausea. These are not mutually exclusive but the efficacy of drugs targeted at sites **A** and **B** will differ depending upon which mechanism is in operation. In **hypothesis A** (left hand panel) the activation of central emetic pathways activates ascending pathways leading to the sensation of nausea, followed by descending autonomic pathways leading to delayed gastric emptying. An anti-nausea drug targeted centrally (site **A**) would block both nausea and the peripheral motility changes, so there will be a secondary return of gastric emptying to normal. In this hypothesis a drug targeted at site **B** may only have a small effect by reducing a positive reinforcing feedback from the centrally-driven disruption of motility. In **hypothesis B** (right hand panel) disordered upper digestive tract motility, usually resulting from disease (e.g., diabetic gastroparesis), is the primary driver for the genesis of nausea, leading to activation of visceral afferents or possibly the release of enteroendocrine agents into the blood for subsequent activity at the area postrema. A drug acting on the upper digestive tract (site **B**) would normalize gastric motility and remove the primary drive for nausea. Note that in this hypothesis, the “traditional prokinetic drugs” (with an exclusively peripheral action) have generally not been successful; potential alternatives are indicated. In this hypothesis a drug acting at the central site **A** would also be likely to indirectly reduce nausea by preventing activation of central pathways. ENS, enteric nervous system; ICC, interstitial cells of Cajal.

Among the antipsychotic compounds (including the butyrophenone haloperidol, discovered in 1958 by Paul Janssen; Sneader, 2005) Janssen Pharmaceutica (Tables 1, 2) developed in the mid-1950s, some were effective antagonists at the dopamine receptors in the AP involved in induction of vomiting. Since this region of the brain has a relatively permeable blood-brain barrier, a search was made for antagonists that did not cross this barrier and hence, were less likely to evoke extrapyramidal side-effects caused by antagonism of dopamine receptors within the brain. Using the now-established model of apomorphine-induced emesis in dogs, domperidone was identified in 1974 from the butyrophenone class of molecules. The drug was erroneously described as similar to metoclopramide (Champion et al., 1986; perpetuating the belief that all of the actions of metoclopramide must be due to antagonism of the effects of dopamine) and marketed in 1982 (Champion et al., 1986; Barone, 1999) for prevention of nausea and vomiting (Figure 7) including that induced by anti-cancer chemotherapy, then as a gastroprokinetic agent (Ahmad et al., 2006) and galactogogue. Later studies showed that domperidone has a similar affinity for the human D_2_ and D_3_ receptors (Ki-values, respectively, 12.6 and 4 nM^5^), no ability to interact with the 5-HT_4_ receptor but at slightly higher concentrations acts as a α_1_-adrenoceptor antagonist (Ki of 71 nM: Keiser et al., 2009; see also Ennis and Cox 1980; Ison and Peroutka, 1986).

Investigation of the utility of dopamine receptor antagonists as anti-emetics continues with investigations of other D_2_/D_3_ receptor antagonists, such as amisulpride (Kranke et al., 2013) and ATC-1906^6^, aiming primarily to achieve an improved safety profile over domperidone (i.e., its cytochrome P450 interaction liability and occasional reports of prolongation of cardiac QTc intervals; Ortiz et al., 2015) and gain access to patients in the USA (where domperidone is not registered) as well as the rest of the world, for treatment of gastroparesis.

### Dexamethasone; a synthetic glucocorticoid

Baker et al. (1979) found that dexamethasone (10 mg) reduced vomiting caused by different cytotoxic anti-cancer drugs but it was suggested that the associated euphoria played a role. A pilot study using methylprednisolone to inhibit prostaglandin release (Rich et al., 1980) also showed efficacy (in combination with chlorpromazine or prochlorperazine) in patients receiving cisplatin-based therapy. Later studies using high-dose dexamethasone in patients receiving cisplatin alone or in combination with other cytotoxic drugs reported impressive responses with excellent or good control of nausea and vomiting in 50% of patients who had failed on standard anti-emetics and 71% in patients who had not received anti-emetics previously (Aapro and Alberts, 1981). Notably, synthetic corticosteroids do not inhibit the acute, rapid-onset forms of vomiting induced by apomorphine or ipecacuanha (Axelsson et al., 2003; Sam et al., 2003), suggesting involvement of an “inflammatory” component in the mechanisms of chemotherapy-induced emesis (Sanger and Andrews, 2006).

Although now widely used in combination with other anti-emetic drugs the mechanism and site of action is still not clear. One suggestion is that dexamethasone may supress eicosanoid metabolism, inflammation and edema induced by chemo-radiotherapy (Andrews and Rudd, 2016; see Chu et al., 2014 for review).

### Cannabinoids

In the early 1970s, anecdotal reports emerged of reduced nausea and vomiting by marijuana-users undergoing chemotherapy for Hodgkin's disease, leading to clinical evaluation of the anti-emetic use of marijuana and THC (Δ-9-tetrahydrocannabinol, the major psychoactive constituent) in cancer patients receiving chemotherapy (Sallen et al., 1975; Vincent et al., 1983; Parker et al., 2011). Thereafter, the Food and Drug Administration (FDA) was recommended by the Oncologic Drug Advisory Committee to classify THC for use against refractory chemotherapy-induced emesis (Vincent et al., 1983). Cannabinoids (THC, nabilone, levonantrodol) were extensively investigated as anti-emetics in anti-cancer chemotherapy in the late 1970s and early 1980s with a 1981 survey indicating THC inclusion in 26.5% of studies, intermediate between prochlorperazine (41.2%) and metoclopramide (20.6%; Penta et al., 1981). Although cannabinoids were superior to placebo and prochlorperazine, they were not pursued at the time because of side-effects and probably also because of the discovery of the anti-emetic efficacy of 5-HT_3_ receptor antagonists a few years later (see below).

Developments in cannabinoid receptor pharmacology and the availability of selective ligands prompted a resurgence of interest in the anti-emetic effects of cannabinoids (Darmani, 2001; Simoneau et al., 2001) and particularly their potential in treatment of chemotherapy-induced nausea (Rock and Parker, 2016). These agents have been shown to be effective against vomiting and behaviors suggestive of nausea (see below) in several animal models (ferret, least shrew, house musk shrew, rat). In contrast to other agents discussed above, they act as a receptor agonist, activating CB_1_ receptors in the dorsal vagal complex of the brainstem (Van Sickle et al., 2003) and the visceral insular cortex (Limebeer et al., 2012). The clinical potential of the selective CB_1_ receptor agonists remains to be evaluated.

## The 1980s: a new era in control of nausea and vomiting prompted by changes in chemotherapy

The rise in treatment of cancer from the 1960s to 1980 also saw an increase in the number of anti-emetic studies in cancer patients. From 1963 such studies increased from 1 to 12 *per annum* in 1980 involving 25 different compounds alone or in combination (Penta et al., 1981). An important driver was the introduction of more effective, but unfortunately more emetic, chemotherapy agents and in particular cisplatin, in 1971 (for history of platinum salts, see Christie and Tansey, 2007). The limited efficacy of anti-emetic drugs in these new therapeutic regimes prompted research which led to the discovery of 5-HT_3_ receptor antagonists, discussed below. A clinical study published in 1984 (Plezia et al., 1984) reported that acute vomiting induced by cisplatin-containing treatments could be blocked by an “intensive five drug regime” (metoclopramide, diphenhydramine, dexamethasone, diazepam, thiethylperazine); by 1988 it was possible to achieve the same effect by intravenous injection of a 5-HT_3_ receptor antagonist alone (Cassidy et al., 1988). Although the introduction of cisplatin was a significant stimulus for research into novel anti-emetic drugs it should not be forgotten that radiation was also used to treat cancer and also given prior to bone marrow transplantation, causing severe nausea and vomiting (Danjoux et al., 1979); as late as 1978 general anesthesia was being used to prevent acute vomiting resulting from total body irradiation (Whitwam et al., 1978).

### 5-hydroxytryptamine_3_ receptor antagonists

Gylys et al. (1979) found that in conscious dogs, metoclopramide more effectively inhibited vomiting evoked by cisplatin, compared with chlorpromazine, haloperidol, domperidone, or nabilone. Then in 1981, high intravenous doses of metoclopramide were shown to reduce emesis in patients receiving cisplatin for treatment of cancer, contrasting with the poor effectiveness of prochlorperazine (Gralla et al., 1981). The rationale for using the high dose was later explained by Gralla (Christie and Tansey 2007). In brief, they realized that the phenothiazines and the cannabinoids were not working well so they needed another approach. In the USA, metoclopramide was still a relatively new drug (it was widely used in Europe) and since the dose was not well-established for the indication of emesis it was decided to undertake a trial that escalated the dose to maximize the chance of success. As Gralla recalled “I looked at the world's suicide literature and it looked as though it was impossible to kill yourself with the drug, so that sounded good.” Following the successful use of high-dose metoclopramide later trials failed to replicate this activity with high doses of the D_2_ receptor antagonists domperidone (no change in protection but serious side-effects noted; Tonato et al., 1985) and alizapride (less effective than metoclopramide and caused severe hypotension; Saller and Hellenbrecht, 1985). Thus, it began to seem unlikely that high doses of metoclopramide achieved greater anti-emetic activity simply because it somehow blocked D_2_ receptors in the brain more effectively. At that time, one possibility was that the ability of metoclopramide to increase gastric emptying may in some way supplement the anti-emetic activity of this drug by accelerating emptying of the stomach thus overcoming the gastric stasis which accompanies nausea and precedes vomiting (see Figure 7).

The anti-emetic activity of metoclopramide was confirmed by use of a ferret model of emesis to demonstrate efficacy against different chemotherapeutic agents. The model was introduced by Floczyk et al. (1982) using cisplatin as the emetic stimulus, confirmed by Miner and Sanger (1986) and quickly extended to study the effects of the chemotherapeutic drugs doxorubicin and cyclophosphamide (Schurig et al., 1984; Miner et al., 1987) and whole body irradiation (Gylys and Gidda 1986; Andrews and Hawthorn 1987; Miner et al. 1987). The history of the use of the ferret in anti-emetic research is reviewed Percie du Sert and Andrews (2014) and this model has largely supplanted the use of dogs as the first species in which novel anti-emetics are studied and as a species for investigating emetic potential of NCEs.

Research within Beecham Pharmaceuticals (Figure 2; Table 1) using ferrets showed that cisplatin-induced emesis was unaffected by domperidone but prevented by renzapride (BRL24924), a molecule originally identified as a potent stimulant of gastric motility (and an agonist at the “myenteric-like 5-HT receptor” or 5-HT_4_; see above) without ability to antagonize at the D_2_ receptor (and subsequently shown to potently antagonize at the 5-HT M or 5-HT_3_ receptor; Miner et al., 1986, 1987; Sanger, 1987a). Since these experiments could not rule out the possibility that anti-emetic activity was achieved by stimulation of gastric emptying alone (Alphin et al., 1986) it was necessary to perform additional experiments with the recently described selective 5-HT_3_ receptor antagonist MDL72222 (a generous gift to G.J. Sanger from J.R. Fozard, then at Merrel-Dow). The resultant complete control of vomiting demonstrated for the first time, that powerful anti-emetic activity could be achieved by 5-HT_3_ receptor antagonism alone (Miner et al., 1986).

Prior to these studies in ferrets it had become clear that metoclopramide could also interact with 5-HT receptors which were, at the time, poorly understood. The drug antagonized a neuronally-mediated action of 5-HT in guinea-pig isolated colon and ileum (Bianchi et al., 1970; Birtley and Baines, 1973; Bury and Mashford, 1976; Fozard and Mobarok Ali, 1978), defining metoclopramide as a 5-HT M receptor antagonist. Metoclopramide could also antagonize other neuronally-mediated actions of 5-HT in the peripheral nervous system, most notably, 5-HT-evoked tachycardia in rabbit isolated heart or bradycardia in anesthetized rats (the von Bezold-Jarisch reflex; Fozard and Mobarok Ali, 1978; Fozard, 1983). Fozard and colleagues subsequently showed that (–)-cocaine and structurally-related compounds also antagonized these actions of 5-HT, leading to synthesis of MDL72222 from the chemical template of cocaine by Merrell Dow (Figure 2; Tables 1, 2), the first selective 5-HT_3_ receptor antagonist, then aimed at treatment of migraine (Fozard, 1984).

The anti-emetic experiments, conducted in the laboratories of Beecham Pharmaceuticals, were quickly replicated using their own compound (the selective 5-HT_3_ receptor antagonist BRL43694 or granisetron; Boyle et al., 1987; Bermudez et al., 1988) and those from their competitors including: Glaxo (GR38032F or ondansetron, a racemate designed for “a variety of disorders including migraine” before being specifically patented for treatment of depression, schizophrenia, anxiety and cognitive disorders^7^); Sandoz (ICS 205-930 or tropisetron, designed for treatment of migraine and later found to have some ability to antagonize at the 5-HT_4_ receptor); and Merrell Dow (MDL72222 or bemesetron, for treatment of migraine). These studies led to the filing of a patent claiming the use of these compounds for treatment of emesis Sanger and Miner 1988, successfully upheld over ondansetron (Cavella et al., 1997, p. 27). Significantly, anti-emetic efficacy was not just restricted to the control of cisplatin-induced-emesis but was equally effective against different chemotherapeutic drugs (Miner et al., 1987). Further, emesis could be controlled even after it had begun (Miner et al., 1987), later of great importance in positioning the 5-HT_3_ receptor antagonists as both prophylactic treatments and for control of breakthrough emesis. An additional control experiment, required at the time, was to demonstrate that 5-HT_3_ receptor antagonism by granisetron did not also prevent the anti-tumor activity of cisplatin (Goddard et al., 1990). There was now no doubt that the experiments within the Beecham Laboratories had demonstrated the role of the 5-HT_3_ receptor in the mechanisms by which chemo- and radio-therapy evoke nausea and vomiting (reviewed in Sanger, 1990).

During this time and following the original abstract highlighting the anti-emetic activity of renzapride (Miner et al., 1986), experiments to demonstrate the anti-emetic activity of the 5-HT_3_ receptor antagonist ICS 205-930 (Costall et al., 1986) were swiftly sponsored by Sandoz, the manufacturer of ICS 205-930 (see Christie and Tansey 2007). With respect to ondansetron and tropisetron, these can therefore be regarded as examples of “re-purposing” (bemesetron was not progressed for treatment of emesis, the company preferring its follow-up molecule MDL73147 or dolasetron; see Kirchner et al., 1993).

5-HT_3_ receptor antagonists prevent cytotoxic-associated vomiting by blocking the ability of 5-HT, released from mucosal enterochromaffin cells in the upper GI tract, to activate 5-HT_3_ receptors on abdominal vagal nerve terminals and thereby “desensitize” the vagus to the pro-emetic stimulatory actions of 5-HT and other substances (e.g., prostanoids) released during the cytotoxic treatment (Andrews et al., 1988; see Andrews and Rudd, 2016 for review of more recent evidence).

The more advanced stage of clinical and safety testing of ondansetron (for CNS disorders) meant that this drug was first to achieve registration by the FDA in 1991, followed in the same year by granisetron in other countries and in particular, by Japan in 1992. Later, there would be controversy over the number of published clinical trials reported for ondansetron, which appeared to have been reported more than once under different authorship in different publications (Rennie, 1999), calling for registration of clinical trials (now best practice). Nevertheless, today, selective 5-HT_3_ receptor antagonists are an essential component of anti-emetic therapy in patients undergoing chemotherapy and together with the NK_1_ receptor antagonists (see below) has revolutionized treatment of cancer and reduced health care costs (Currow et al., 1997; Warr and DeAngelis, 2009).

### Neurokinin_1_ (NK_1_) receptor antagonists

The widespread clinical use of 5-HT_3_ receptor antagonists to treat chemotherapy-induced nausea and vomiting (CINV) and to a lesser extent PONV, established the clinical need and hence, the market value of an anti-emetic drug, which could exceed one billion $US *per annum*, further stimulating interest in this therapeutic area. Additionally, the primary efficacy of 5-HT_3_ receptor antagonists in the acute phase of highly emetic chemotherapy (e.g., cisplatin containing regimes) as compared to the delayed phase where they appeared less efficacious, their lower efficacy against nausea as compared to vomiting for both CINV and PONV, and their lack of effect against emesis induced by motion and apomorphine, illustrated the need for further developments.

Substance P was identified by von Euler and Gaddum in 1931; the name originates from the phrase in their paper “This standard preparation, which we call P….” (von Euler and Gaddum, 1931, p. 80). Over the last 40 years research into the actions of substance P has been most closely associated with pain pathways with focus on the neurokinin_1_(NK_1_) receptor as the primary receptor for substance P in mammals (see Borsook et al., 2012). Studies, largely in rodents, identified non-peptide small molecules acting as antagonists at the NK_1_ receptor for potential clinical use as analgesics. During this time, the involvement of substance P (or other tachykinins) in mechanisms of nausea and vomiting was largely overlooked, despite a body of literature summarized in Table 4, which in many ways parallels that for its involvement in pain (see Andrews and Rudd, 2004). Definitive evidence for the involvement of substance P in emesis in animals came only with the development of the non-peptide, brain penetrant, NK_1_ receptor antagonists disclosed by Pfizer (CP-96,435, Snider et al., 1991; CP-99,994, McLean et al., 1993). The first published studies showing anti-emetic effects were in the ferret by researchers at Glaxo (Bountra et al., 1993; Gardner et al., 1994) and Merck (Tattersall et al., 1993, 1994) but using a Pfizer compound (CP-99,994). These were followed by a detailed study in the ferret, cat, house musk shrew and dog from Pfizer with academic colleagues (Watson et al., 1995a,b). Overall the studies demonstrated that NK_1_ receptor antagonists had a different profile from 5-HT_3_ receptor antagonists (and muscarinic and H_1_ receptor antagonists) in their ability to block both acute and delayed cisplatin-induced emesis, to block emesis induced by both peripherally (e.g., copper sulfate, abdominal vagal afferent electrical stimulation) and centrally-acting stimuli (e.g., morphine, apomorphine) and also to reduce motion-induced emesis. This unique preclinical profile rekindled interest in the area of anti-emetics. However, a major question was whether these encouraging pre-clinical findings (largely from the ferret) would translate to the clinic. This question arose because despite the pre-clinical data (largely from the rat) for the involvement of Substance P in pain pathways, contemporaneous published clinical studies of analgesic effects of NK_1_ receptor antagonists were equivocal (e.g., Dionne et al., 1998; Reinhardt et al., 1998; see Rupniak and Kramer, 1999; Hill, 2000; Borsook et al., 2012 for reviews). Among the suggested reasons for this failure (Laird et al., 2000) was the potential for receptor/neurotransmitter redundancy in pain-conducting systems (e.g., for the NK_1_, NK_2_, NK_3_ receptors small differences in affinity for endogenous ligands meant that “ligand promiscuity” was a real possibility; Maggi, 2000; Sanger, 2004) or a mismatch between the measure of “nociception” in animals and the human sensation of pain.

**Table 4:** A summary of the key pieces of evidence implicating substance P and related tachykinins in emesis.

**Date**	**Evidence**	**Species**	**References**	**Comment**
1936	Substance P (SP) extracted from the vagus	Dog	von Euler, 1936	•The vagus had been implicated in the induction of emesis by early animal studies (Hatcher, 1924) and studied in the 1920s (cited in Lewis, 1942) when induction of nausea was reported in humans by stimulation of the vagus •Subsequent demonstration in the vagus and nodose ganglion of multiple species including human (e.g., Lundberg et al., 1978). Also vagal afferents terminating in the *nucleus tractus solitarius* (NTS) shown to be source of some of the Substance P in the dorsal brainstem (see Andrews and Rudd, 2004)
1951	High levels of SP extracted from the digestive tract mucosa	Dog	Douglas et al., 1951	•Digestive tract mucosa enterochromaffin cells shown to be a rich source of 5-HT in a range of species in the 1950s, accounting for the majority of 5-HT in the mammalian body (Faustini, 1955; Erspamer and Testini, 1959)
1954	High concentrations of SP in the area postrema (AP); Authors comment: “the AP only contains active substances by virtue of its chemoreceptive properties… One of the functions of some parts of this tissue may be to act as a chemoreceptor for substances in the blood stream to convert messages received in this way into nervous impulses.”	Dog	Amin et al., 1954	•Although the AP was implicated in emesis by older papers (e.g., see Hatcher, 1924) the seminal paper by Wang and Borison (1952) highlighted its role as a chemoreceptive region of the brain
1963	Induction of emesis by subcutaneous administration of eledoisin (a tachykinin closely related to SP and extracted from posterior salivary glands of the octopod *Eledone cirrhosa*)	Dog	Erspamer and Glasser, 1963	•The frog skin tachykinin, pysalemin (subcutaneously and intravenously) induced vomiting in the dog Bertaccini et al. (1965) and intravenous SP was shown subsequently to have a similar effect, although so did many other peptides Carpenter et al. (1983, 1984)
1981	Immunohistochemical localization of SP in the AP to varicose processes but absence of SP-positive cell bodies	Rat	Armstrong et al., 1982	•Findings confirmed and extended by Pickel and Armstong (1984) but as rodents lack an emetic reflex (but see text for discussion) the relevance to emesis may have been overlooked. Newton et al. (1985) confirmed and extended the rat finding to the cat, a species with an emetic reflex so potentially of more relevance to humans.
1981	High levels of SP in human brainstem including area postrema	Human	Cooper et al., 1981	•A study in 1955 (i.e., a year after the (Amin et al., 1954) dog study) had found little SP in the human AP and may have led to the view that there were species differences, resulting in dismissal of the potential clinical significance of the dog study.
1983	Activation of AP neurons by ionophoretic application of SP	Dog	Carpenter et al., 1983, 1984	•Electrophysiological evidence for excitatory effects of SP in a relevant species, but numerous other peptides had similar effects, possibly reducing the significance of the observation
1984	Demonstration of high levels of SP receptors in the *nucleus tractus solitarius* and moderate levels in the area postrema	Rat	Helke et al., 1984	•SP-sensitive receptors investigated using [^125^I]Bolton-Hunter SP •NTS implicated in coordination of visceral and somatic motor outputs for emesis and integration of afferent signals prior to projection to more rostral brain regions (see text for details)
1988	Induction of retching in the urethane anesthetized ferret by topical application of SP (0.1 mM) to the fourth ventricle	Ferret	Wood, 1988	•Proposed that the action was either directly on the AP or via access to the dorsal NTS, particularly the *subnucleus gelatinosus*. A subsequent study in conscious ferrets showed that injection of SP into the NTS induced emesis (Gardner et al., 1994)
1992	Acute administration of the ultrapotent capsaicin analog (RTX) to the ferret has anti-emetic effects against both centrally and peripherally acting stimuli	Ferret	Bhandari and Andrews, 1992	•In the subsequent full paper (Andrews and Bhandhari, 1993) it was proposed that “the most likely mechanism to account for the anti-emetic effects is that RTX induces a depletion of a neurotransmitter, possibly substance P or CGRP, at a central site in the emetic pathway”
1993	First preclinical publications showing anti-emetic efficacy of a non-peptide NK_1_ receptor antagonists (CP-99,994)	Ferret	Bountra et al., 1993; Tattersall et al., 1993	•These publications were from scientists at Glaxo and Merck but the compound used (CP-99, 994) was a Pfizer compound (Watson et al., 1995a,b). See text for details of other compounds and discussion of spectrum of anti-emetic effects.
1997	First clinical publication of anti-emetic effects on a non-peptide NK_1_ receptor antagonist (CP-122,721) against high dose cisplatin chemotherapy	Human	Kris et al., 1997	•This study supported the translation of ferret data to human and demonstrated significant efficacy in the delayed phase, in contrast to the effects of 5-HT_3_ receptor antagonists (see text for details)
2003	Approval of Aprepitant (Emend®) by European Medicines Evaluation Agency and Food and Drug Administration for treatment of emesis induced by cisplatin chemotherapy	Human		

*For detailed discussion see Andrews and Rudd (2004)*.

A key issue in increasing the likelihood that data obtained in the ferret would translate was the early recognition of marked species differences in NK_1_ receptor pharmacology with some compounds having a relatively high affinity at the rat receptor compared the human NK_1_ receptor (e.g., RP67580) whereas others had a relatively high affinity at the human compared to the rat receptor (e.g., L743310; see Table 1, p. 382, Andrews and Rudd 2004). Taking CP-99,994 as an example, as it was the compound most widely used in establishing the *in vivo* effects of NK_1_ receptor antagonists, it has relatively high affinity at the human (K_i_ 0.3 nM) and ferret (K_i_ 1.7 nM) NK_1_ receptors in contrast to the rat receptor (K_i_ 111 nM); a similar pattern is found with other NK_1_ receptor antagonists (Andrews and Rudd 2004). *In vitro* autoradiographic studies showed that CP-99,994 displaced [^3^H]-substance P from the ferret brainstem including the AP and the subnucleus gelatinosus region of the NTS in a concentration-related manner over 0.1–100 nM (Watson et al., 1995a). It should be noted that technological advances in brain imaging now make it possible to study ligand-receptor interactions *in vivo* in animals (e.g., Chin et al., 2006) and humans (e.g., Borsook et al., 2012) facilitating compound and clinical dose-selection and hopefully enhancing translation.

The first human study of an NK_1_ receptor antagonist was published in 1997 (Kris et al., 1997), < 4 years after the first pre-clinical publication. This rapid time was facilitated by prior safety studies required for the earlier analgesic studies (see above) and illustrates why progress can sometimes be rapid if a drug has already been investigated in another therapeutic area. In 17 patients undergoing highly emetogenic cisplatin chemotherapy CP-122,721 was efficacious overall but the effect was particularly marked (83% complete control) in the delayed phase of emesis. Further studies in patients undergoing chemotherapy followed, using other compounds (e.g., CJ-11,974, Hesketh et al., 1999; L-54030 and L758298, Navari et al., 1999) and compounds were also investigated for efficacy in PONV (CP-122, 721, Gesztesi et al., 1998; GR-205171, Diemunsch et al., 1999).

Currently, four NK_1_ receptor antagonists are approved for human clinical use: aprepitant, fosaprepritant [intravenous formulation of aprepitant (see Hale et al., 1998, for characterization)], rolapitant, and netupitant, the primary differences being potency and duration of action. The most recent MASCC/ESMO guidelines for high emetic-risk chemotherapy (Herrstedt et al., 2017) recommend use of an NK_1_ receptor antagonist in combination with a 5-HT_3_ receptor antagonist and dexamethasone for optimal efficacy.

It is worthwhile noting that the NK_1_ receptor antagonist maropitant (Benchaoui et al., 2007b) is marketed (Cerenia™) for prevention of acute vomiting in dogs. It has been used for treatment of vomiting in dogs undergoing cisplatin-chemotherapy (Vail et al., 2007) but also has efficacy against vomiting in other indications including parvoviral enteritis and pancreatitis (de la Puente-Redondo et al., 2007) as well as blocking vomiting induced by hydromorphone when used as a surgical premedication (Claude et al., 2014) and motion sickness (Benchaoui et al., 2007a). Maropitant is available for prevention of vomiting in cats (Batchelor et al., 2013). Other anti-emetics used in humans such as metoclopramide and ondansetron have also found veterinary use (Kenward et al., 2017).

A final note: Among all the proposed clinical indications for NK_1_ receptor antagonists (especially pain, depression, anxiety, emesis), based on animal and human data (Kramer et al., 1998; Saria, 1999), only the anti-emetic indication successfully translated to clinical usage. For emesis at least, this activity was not subject to putative “promiscuity” among NK receptors for endogenous ligands (see above); NK_3_ receptor antagonism did not inhibit cisplatin-evoked emesis in ferrets (King and Sanger, 2005).

### NK_1_ and 5-HT_3_ receptor crosstalk

Palonosetron (RS 25259-197) was synthesized and characterized by Syntex Discovery Research (Clark et al., 1993; Eglen et al., 1995), before being licensed to Eisai and Helsinn for co-marketing in the USA in 2003 (the same year as aprepitant was approved by the EMEA and FDA). The drug has a relatively high binding affinity for the 5-HT_3_ receptor (Wong et al., 1995; Muchatuta and Paech, 2009) and a long plasma half-life in healthy volunteers (Stoltz et al., 2004; Muchatuta and Paech, 2009). Surprisingly, palonosetron was effective in both acute and delayed phases of CINV. The drug did not antagonize the NK_1_ receptor (Wong et al., 1995) and since other 5-HT_3_ receptor antagonists did not have the same efficacy profile, research was initiated to explain these findings. This showed that in contrast to the first generation of 5-HT_3_ receptor antagonists, which are competitive receptor antagonists, palonosetron binds allosterically to the receptor, exhibiting positive cooperativity; the authors argued that the difference in structure between palonosetron and the earlier 5-HT_3_ receptor antagonists may, somehow, explain this difference (Rojas and Slusher, 2012). Further experiments demonstrated a persistent ability to inhibit receptor function after the drug was removed, triggering receptor internalization of the drug-receptor complex into the cell (Rojas et al., 2010). Since palonosetron remained bound to the 5-HT_3_ receptor, this internalization now persisted for much longer than anticipated for a simple competitively-acting receptor ligand, raising the possibility that the internalized complex could interact and “crosstalk” with NK_1_ receptor signaling pathways, inhibiting the functions of substance P (Rojas and Slusher, 2012; Rojas et al., 2014). Furthermore, palonosetron inhibited the upregulation of substance P expression in the nodose ganglia induced by cisplatin in rats, whereas granisetron and other 5-HT_3_ receptor antagonists did not (Rojas and Slusher, 2012).

Interestingly, a possible interaction between 5-HT_3_/NK_1_ receptors had been demonstrated 10 years previously by Minami et al. (2001) using *in vivo* recording from ferret abdominal vagal afferents (e.g., Minami et al., 2001). This study showed that an NK_1_ receptor antagonist (CP-99,994) reduced the afferent response to 5-HT and conversely the 5-HT_3_ receptor antagonist granisetron reduced the afferent response to Substance P.

To date, palonosetron is the only 5-HT_3_ receptor antagonist approved by the FDA for prevention of both acute and delayed CINV. The combination of palonosetron with NK_1_ receptor antagonists such as netupitant therefore appears to have synergistic activity and good efficacy against both “acute” and “delayed” emesis (Rojas et al., 2014). Indeed, when these two drugs are given together with dexamethasone, total control of cisplatin-induced vomiting has been reported in the absence of significant nausea (Aapro et al., 2014; Keating, 2015). Today, Helsinn markets an oral fixed-dose combination product of netupitant with palonosetron (NEPA) for prevention of CINV.

The experience with palonosteron demonstrates that the pharmacological profile of a compound defined at the time of discovery does not necessarily predict the *in vivo* effects.

## Challenges in identification of novel anti-emetic drugs

### No single organ target

Nausea and vomiting involve multiple organs and systems (e.g., visceral and somatic divisions of the peripheral nervous system, the digestive tract and respiratory system), including the central nervous system (CNS) which integrates the sensory inputs and motor outputs. Thus, there is no obvious single physiological pathway or organ to study, in contrast to asthma (airways), peptic ulcer (gastric and duodenal mucosa) and angina (coronary circulation). Pain, with sensory, behavioral, CNS, and motor components would be the most analogous clinical problem to nausea and vomiting.

The lack of a clear “target organ” means that it is difficult to apply modern molecular techniques for target identification and validation, and such methods have not (yet) contributed to anti-emetic drug discovery. Nevertheless, twin and (Reavley et al., 2006) genome-wide association studies (Hromatka et al., 2015) of motion sickness begin to illustrate the potential for molecular studies to provide insights into tractable targets.

### Animal models and their translational value

The commonly-used laboratory rodent species do not vomit (Sanger et al., 2011; Horn et al., 2013) so most early research used non-human primates (particularly the squirrel monkey in motion sickness research) and dogs, with a few studies utilizing cats. Although dogs have been used for emesis research for at least 150 years (see Hatcher and Weiss, 1923 for review of early literature), in the last 35 years ferrets and to a lesser extent mink (both carnivores) have largely supplanted dogs for emesis research (see Percie du Sert and Andrews, 2014 for review of the history of their use in emesis research and references) although cats continue to be used for studies of motion sickness (e.g., Yates et al., 2014). The insectivore *Suncus murinus* (house musk shrew) has also been utilized, largely because it is highly sensitive to motion (Ueno et al., 1987, 1988) and its small size (< 100 g) reduces the amount of a novel compound that needs to be synthesized for testing *in vivo*. Similarly, the least shrew (*Cryptotis parva*) which only weighs ~5 g has also been utilized (e.g., Zhong et al., 2014). However, for most of these species their genome has not been sequenced, hampering translation of receptor pharmacology across species. It is also important to note that for an animal model to have translational value for humans, the species must respond to the same stimulus (preferably at doses comparable to those used clinically), must cause emesis by the same pathway/mechanism as in humans (bearing in mind that pathways may exhibit plasticity as the result of disease and the mechanism in humans may not be known) and must involve the same neurotransmitter and receptor sub-type in the pathway.

A critical question related to translation is “*Do Animals Experience Nausea and if so, How could it be Measured?”* The mechanical act of vomiting is broadly similar in humans and the laboratory animals. Until relatively recently, the ability of a substance to block retching and vomiting in an animal was taken as an indication that nausea was also likely to be blocked when tested in humans. For example, as some behaviors accompanying cytotoxic drug-induced emesis in ferrets were inhibited by 5-HT_3_ receptor antagonists (e.g., burrowing and backing-up movements; Bermudez et al., 1988; Hawthorn and Cunningham, 1990; but see Lau et al., 2005a,b for more recent analysis) it seemed reasonable to suggest that 5-HT_3_ receptor antagonists could also have anti-nausea effects in humans. However, it has since become apparent that 5-HT_3_ receptor antagonists have a relatively lower efficacy against nausea induced by chemotherapy as opposed to vomiting (Soukop, 1990). Research in animals continues (there is considerable debate regarding nausea in animals and the nature of the assumed sensory experience) and many pre-clinical studies investigating mechanisms of emesis now include one or more of the measurements argued to be indices of nausea (e.g., Horn et al., 2011; Lu et al., 2017a,b; for detailed discussion of the issues see Stern et al., 2011, Chapter 8; Andrews and Sanger, 2014). Additionally, in animals, *post mortem* analysis of the pattern of activation of brain nuclei indicated by *c-Fos* immunohistochemistry can also give insights into which “higher” brain regions can be activated by an emetic, giving some insight into possible sensory experiences which may accompany vomiting and/or nausea (Lu et al., 2017b; Tu et al., 2017).

### The challenges of research on nausea and vomiting in humans

Studies of the physiology and pharmacology of nausea and vomiting in healthy volunteers are not common and usually involve use of relatively mild stimuli so only nausea is induced. These include apomorphine (Isaacs and MacArthur, 1954), ipecacuanha (Minton et al., 1993), and opioid receptor agonists (Soergel et al., 2014) together with motion, most commonly in the form of vection.

Although clinical trial design methodology is well-established, improved methodology for real-time, more objective and quantitative measurements of nausea and vomiting is needed in humans to improve characterization of the side effects of new treatments and better characterize the effects of anti-emetics, also facilitating more valid comparisons with pre-clinical studies.

Four areas appear promising for human research: (i) Improved understanding of the neuropharmacology of brain pathways implicated in nausea using brain imaging techniques combined with physiological studies of changes accompanying nausea (e.g., heart rate variability, plasma vasopressin and gastric motility); (ii) Analysis of large patient data sets to identify relationships between symptoms (e.g., nausea, early satiety) and underlying pathology (e.g., dysfunctional interstitial cells of Cajal within the stomach wall; see below); (iii) Precise characterization of the efficacy of anti-emetics in specific patient sub-populations so that molecular correlates can be identified (e.g., 5-HT_3B_ receptor gene (Tremblay et al., 2003) and ABCB1 polymorphisms (Babaoglu et al., 2005; Tsuji et al., 2013) as predictors of 5-HT_3_ receptor antagonist efficacy in CINV), potentially providing data to develop personalized therapies; (iv) Identification of the genomic/molecular basis for individual and population differences in sensitivity to different emetic stimuli; for example for motion sickness, which itself is a prognostic indicator for other causes of emesis (e.g., Warr, 2014), there are inter-individual (e.g., twin studies, Reavley et al., 2006), sex (female sensitivity > male, Lentz and Collins, 1977) and ethnic (greater sensitivity in Chinese subjects compared with African-American and Caucasian subjects, Stern et al., 1983) differences. Genome-wide association studies of large populations (>80,000 subjects) have begun to identify single nucleotide polymorphisms (SNP) associated with increased motion sickness sensitivity (Hromatka et al., 2015) and SNPs in the opioid receptor gene (*OPRM1*) have been associated with PONV (Sugino et al., 2014).

## Current and future directions in research: lessons from the past for the future

### Repurposing: old drugs for new treatments

A number of the drugs described above were not “designed” as anti-emetics; this was discovered after their introduction for different therapeutic uses. More recently, there is now a growing list of other drugs which were originally used to treat psychosis and depression, and which have subsequently been shown to inhibit nausea and vomiting in several difficult-to-treat indications, including patients receiving palliative care. These include amitriptyline, levomepromazine, mirtazapine, olanzapine, and gabapentin. Table 5 summarizes their discovery, original use approved by the FDA, the key pharmacology and their additional, “repurposed” use as anti-emetic drugs.

**Table 5:** Summary of key drugs “repurposed” for the control of emesis.

**Discovery**	**Original use**	**Summary of pharmacology**	**Anti-emetic use**
**Amitriptyline**				
•Discovered by several groups in 1960^1^	•Tricyclic antidepressant; approved by the FDA in 1961^2^	•Inhibits 5-HT and noradrenaline transporters •Also has affinity for the H_1_ receptor, muscarinic receptors, the α_1_-adrenoceptor and 5-HT_2A_ receptor, at concentrations similar to those which bind 5-HT and noradrenaline transporter sites^4^	•Reduced symptoms in patients with chronic nausea and vomiting (with pain as a predominant symptom) and in diabetic patients with unexplained vomiting^4, 5^
**Levomepromazine**			
•Originally described by Rhone-Poulenc in 1956^6^	•Phenothiazine neuroleptic drug	•Can antagonize at H_1_, muscarinic M_1_/M_2_, D_1_, D_2_, D_3_ and D_4_, receptors, the α_1_ adrenoceptor and the 5HT_2_ receptor^7, 8^	•Has found use in treatment of patients with intractable nausea and vomiting receiving palliative care where it is also used to treat severe delirium or agitation at the end of life^9^
**Mirtazapine (Org 3770)**				
•Synthesized in 2000^10^	•Antidepressant drug	•An antagonist at H_1_, alpha_2_ adrenoceptor, 5-HT_2C_, 5-HT_2A_ and 5-HT_3_ receptors^11^ •Has affinity for several GPCRs, but has highest measurable affinity for α_2_-adrenoceptors (IC_50_ order of potency: _2A_ > _2C_ > _2B_) and 5-HT_2A_ and 5-HT_2C_ receptors (Ki order of potency 5-HT_2C_ > _2A_ > _7_ > _1A_ (Table 2).	•Several case reports and small studies suggest anti-emetic efficacy in patients undergoing surgery, suffering from hyperemesis gravidarum, chronic unexplained nausea and vomiting, and severe gastroparesis unresponsive to conventional treatments^12, 13, 14^ •Also used to treat vomiting and co-morbid anxiety or depressive disorders in patients with chronic or cyclical vomiting syndromes^15^
**Olanzapine**				
•A thienobenzodiazepine originally described by Eli Lilly in 1980^16^	•Atypical antipsychotic	•Has affinity for M_1_, 5-HT_2A_, 5-HT_2B_, 5-HT_2C_, M_4_, H_1_ > M_3_, M_2_, D_2_ > D_4_, D_1_, α_1_-adrenoceptor >5-HT${}_{3}^{17,18,19}$	•Used to prevent and treat breakthrough chemotherapy-induced emesis when given alone and in combination with other anti-emetic drugs^20^ including patients receiving palliative care^21, 22^ •For example, a significant improvement in nausea reported when given together with 5-HT_3_ and NK_1_ receptor antagonists^23, 24^
**Gabapentin**				
•Synthesized in 1974 (by Gerhard Satzinger) at Parke-Davis (now owned by Warner-Lambert/Pfizer) as potential epilepsy drug, incorporating γ-aminobutyric acid into a lipophilic cyclohexane ring to cross the blood-brain barrier	•Approved by the FDA in 1994 to control partial seizures and in 2002 for conditions with neuropathic pain^25, 26^	•No mechanistic studies in emesis but its analgesic effects are attributed to blockade of the α_2_/δ subunit of voltage-gated calcium channels^27^	•First reported as a potential drug to treat nausea in CINV in 2003 and subsequent studies have extended this to PONV and possibly hyperemesis gravidarum^27, 28^

*^1^Sneader, 2005; ^2^Fangmann et al., 2008; ^3^Owens et al., 1997; ^4^Prakash et al., 1998; ^5^Sawhney et al., 2007; ^6^Sigwald et al., 1956; ^7^Lal et al., 1993; ^8^Srivastava et al., 2009; ^9^Dietz et al., 2013; ^10^Kennis et al., 2000; ^11^Anttila and Leinonen, 2001; ^12^Hasler, 2016; ^13^Kim et al., 2006; ^14^Kundu et al., 2014; ^15^Coskun and Alyanak, 2011; ^16^Chakrabarti et al., 1980; ^17^Bymaster et al., 1996; ^18^Navari, 2014; ^19^Leggio et al., 2016; ^20^Chiu et al., 2016; ^21^Atkinson, 2014; ^22^MacIntosh, 2016; ^23^Hocking and Kichenadasse, 2014; ^24^Navari, 2014; ^25^https://www.chemistryworld.com/podcasts/gabapentin/1017577.article; ^26^Sirven, 2010; ^27^Guttuso et al., 2003; ^28^Guttuso, 2014*.

### Nausea: old and new approaches (table 3)

Nausea still remains relatively poorly treated in comparison to vomiting in many clinical settings including CINV (e.g., Jordan et al., 2016; Aapro, 2018) and there is an increasing recognition in the literature of its importance as a symptom (Donovan et al., 2016); a recent review on CINV posed the question “*Time for more emphasis on nausea?*” (Ng et al., 2014).

### Gastric emptying as a target

Delayed gastric emptying can occur in diverse disorders (e.g., chronic renal failure and Parkinson's), but particularly those with a digestive tract etiology (e.g., gastroparesis, functional dyspepsia, scleroderma) in which nausea is also a symptom. Although evidence for a causal relationship between the genesis of nausea and delayed gastric emptying is inconsistent (Sanger and Pasricha, 2017) there has been a widely held (but also challenged, Sanger and Andrews, 2006; Sanger et al., 2013) assumption since the 1960s that restoring gastric emptying will alleviate the nausea (see McRitchie et al., 1984 for review); this forms the rationale for preferential use of prokinetic (and also anti-emetic) drugs such as metoclopramide (the longest approved drug for treatment of gastroparesis; Schulze-Delrieu, 1979; Camilleri et al., 2013) and domperidone (Brogden et al., 1982) to alleviate nausea (Figure 7). This approach has been pursued more recently by exploiting the gastric prokinetic properties of the antibiotic drugs erythromycin and azithromycin (Broad and Sanger, 2013), providing another example of “repurposing” and stimulating research into the prokinetic effects of macrolides (Broad et al., 2012). Nevertheless, until the precise mechanistic relationship between the various causes of delayed gastric emptying (e.g., disruption of the ENS, e.g., diabetic enteric neuropathy, Chandrasekharan and Srinivasan, 2007) and nausea is understood in a range of disorders, approaches based on prokinetics will continue to be more empirically, rather than rationally based.

### Gastric dysrhythmia as a target

Movements of the human stomach muscles are regulated or “paced” by interstitial cells such as the interstitial cells of Cajal (ICC) which exist as different syncytia within the stomach wall (e.g., Rhee et al., 2011). In summary, these cells generate electrical slow waves which are transmitted to smooth muscle via gap junctions to create “waves” of muscle contraction that move from the gastric corpus down to the pyloric regions, promoting gastric emptying into the intestine (Blair et al., 2014). Increased understanding of their role in the etiology of gastric dysrhythmias linked to nausea (in which changes in functions lead to disrupted patterns of movements within different ICC/muscle syncytia without necessarily changing rates of gastric emptying; see Sanger and Pasricha 2017), particularly in conditions such as gastroparesis (Owyang and Hasler, 2002; Angeli et al., 2015), makes them an increasingly attractive target. The ion channels modulating functions of these cells (Lees-Green et al., 2011) are of particular interest as drug targets.

### Appetite and nausea relationship

Most recently, research has focussed on the concept that nausea might be reduced by drugs which promote appetite, particularly as nausea, vomiting, pain, early satiety and bloating are a common symptom cluster in upper digestive tract disorders such as chronic dyspepsia and gastroparesis (Revicki et al., 2009). The hormone ghrelin has been shown to alleviate anorexia and vomiting in animal models and reduce cachexia and nausea in cancer patients, activities thought to be related to its ability to promote appetite (and perhaps “hedonistic eating” via a constitutively-active receptor; see Sanger and Furness, 2016). In two Phase II studies in patients with diabetic gastroparesis the ghrelin receptor agonist relamorelin, accelerated gastric emptying and reduced vomiting frequency and severity (Lembo et al., 2014, 2016).

### Central nervous system pathways as a target

The sensation of nausea requires activation of pathways in the cerebral hemispheres and most likely the cerebral cortex (Farmer et al., 2015). To block nausea initiated by activation of one of the classical input pathways (vestibular system, area postrema, vagal afferents) will require a drug which acts at some point along the pathway at which these outputs converge to project information to the cerebral hemispheres. The closer the drug acts to the cortical site of sensation genesis the greater will be the probability of treating nausea irrespective of the cause (including psychogenic). Although conceptually simple in approach we currently lack sufficiently detailed knowledge of the pathways in humans activated during nausea and their associated neurotransmitters and receptors.

## Conclusion

The key steps in the identification and development of the current armamentarium of anti-emetic drugs reveal a number of recurrent themes with resonance in other drug discovery areas. These include: the use of traditional medicines as a basis for new drugs; the frequent role of serendipity and exploitation of fortuitous observations; the impact of “non-research” issues such as mergers, takeovers, management decisions, patents, and associated litigation; the challenges of translation of animal models to the clinic in an area where there is no single target organ or molecular mechanism; the advances in pharmacology which change the binding profile and nature of receptor interactions of a drug (even after licensing); the repurposing of drugs active at multiple receptors for one indication but shown subsequently to exert an unanticipated profile of activity in another indication.

The last 30 years since the discovery of 5-HT_3_ and NK_1_ receptor antagonists has seen a major advance in the treatment of nausea and vomiting but major gaps remain including: (a) our understanding of nausea is poor in comparison to pain although it is arguably as common and debilitating, (b) the relationships between appetite, disordered gastric motility and nausea are still not understood, leading to a lack of advances in the treatment of common disorders such as gastroparesis and functional dyspepsia, and (c) there is no registered treatment specifically for nausea irrespective of cause or a “universal anti-emetic,” a drug which would block both nausea and vomiting completely irrespective of the cause (Andrews and Sanger, 2014).

It is notable that the two major breakthroughs (involvement of 5-HT_3_ and NK_1_ receptors) in anti-emetics occurred within < 10 years of each other and in the subsequent >20 years there has not been a comparable “major breakthrough;” why is this the case when molecular pharmacology has exploded over the same period? To some extent this can be explained by the success of the 5-HT_3_ and NK_1_ receptor antagonists in treatment of vomiting, but the same cannot be said for nausea, particularly in conditions such as gastroparesis. We might ask “where will serendipity now occur in an age when the mechanisms of action of drugs are much better understood?” Perhaps an answer can be found in studies looking for single-nucleotide polymorphisms associated with particular patient-symptom associations (e.g., sensitivity to motion sickness; see above).

A comment at a meeting to discuss anti-emetic agents for chemotherapy is pertinent to close: “I believe it is an interesting phenomenon that every 30 years everything done in the past is lost” (Dr. Lassner, p21S, in Penta et al., 1981); perhaps the answer to the question we posed about “where will new anti-emetics come from?” is already there in the recent history of this area.

## Author contributions

All authors listed have made a substantial, direct and intellectual contribution to the work, and approved it for publication.

### Conflict of interest statement

GS is currently in receipt of funding from Takeda Pharmaceuticals, BBSRC together with GlaxoSmithKline, Benevolent and the Dunhill Foundation. He acts as an advisor to Takeda Pharmaceuticals and to Zealand Pharma. He has previously worked for GlaxoSmithKline and its legacy companies (Beecham, SmithKline Beecham), during which he was the designated inventor of the patent for the anti-emetic use of both granisetron and ondansetron. PA has no current conflicts of interest but has in the past acted as a consultant/had research funding from companies whose drugs are discussed in this review (Janssen, SmithKline & French, SmithKline Beecham GlaxoSmith Kline, Pfizer and Merck Sharp & Dohme).

## Footnotes

^1^https://web.archive.org/web/20161213052708/http://www.who.int/medicines/publications/essentialmedicines/EML_2015_FINAL_amended_NOV2015.pdf?ua=1

^2^https://www.asco.org/about-asco/press-center/news-releases/asco-50th-anniversary-poll-names-top-5-advances-past-50-years

^3^http://www.guidetopharmacology.org/

^4^http://www.guidetopharmacology.org/GRAC/LigandDisplayForward?tab=biology&ligandId=83

^5^http://www.guidetopharmacology.org/GRAC/LigandDisplayForward?tab=biology&ligandId=965

^6^https://www.pharmpro.com/news/2016/07/takeda-altos-therapeutics-partner-develop-gastroparesis-treatment

^7^https://www.google.co.ug/patents/US4973594
